# VEGF‐C/VEGFR‐3 axis protects against pressure‐overload induced cardiac dysfunction through regulation of lymphangiogenesis

**DOI:** 10.1002/ctm2.374

**Published:** 2021-03-24

**Authors:** Qiu‐Yue Lin, Yun‐Long Zhang, Jie Bai, Jin‐Qiu Liu, Hui‐Hua Li

**Affiliations:** ^1^ Department of Cardiology, Institute of Cardiovascular Diseases First Affiliated Hospital of Dalian Medical University Dalian China; ^2^ Department of Emergency Medicine Beijing Key Laboratory of Cardiopulmonary Cerebral Resuscitation, Beijing Chaoyang Hospital Capital Medical University Beijing China

**Keywords:** cardiac lymphangiogenesis, heart failure, pressure overload, VEGF‐C, VEGFR‐3

## Abstract

Prolonged pressure overload triggers cardiac hypertrophy and frequently leads to heart failure (HF). Vascular endothelial growth factor‐C (VEGF‐C) and its receptor VEGFR‐3 are components of the central pathway for lymphatic vessel growth (also known as lymphangiogenesis), which has crucial functions in the maintenance of tissue fluid balance and myocardial function after ischemic injury. However, the roles of this pathway in the development of cardiac hypertrophy and dysfunction during pressure overload remain largely unknown. Eight‐ to 10‐week‐old male wild‐type (WT) mice, VEGFR‐3 knockdown (VEGFR‐3^f/−^) mice, and their WT littermates (VEGFR‐3^f/f^) were subjected to pressure overload induced by transverse aortic constriction (TAC) for 1–6 weeks. We found that cardiac lymphangiogenesis and the protein expression of VEGF‐C and VEGFR‐3 were upregulated in the early stage of cardiac hypertrophy but were markedly reduced in failing hearts. Moreover, TAC for 6 weeks significantly reduced cardiac lymphangiogenesis by inhibiting activation of VEGFR‐3‐mediated signals (AKT/ERK1/2, calcineurin A/NFATc1/FOXc2, and CX43), leading to increased cardiac edema, hypertrophy, fibrosis, apoptosis, inflammation, and dysfunction. These effects were further aggravated in VEGFR‐3^f/−^ mice and were dose‐dependently attenuated by delivery of recombinant VEGF‐C_156S_ in WT mice. VEGF‐C_156s_ administration also reversed pre‐established cardiac dysfunction induced by sustained pressure overload. Thus, these results demonstrate, for the first time, that activation of the VEGF‐C‐VEGFR‐3 axis exerts a protective effect during the transition from cardiac hypertrophy to HF and highlight selective stimulation of cardiac lymphangiogenesis as a potential new therapeutic approach for hypertrophic heart diseases.

## Graphical abstract

Schematic illustration for VEGF‐C‐VEGFR‐3 signaling‐mediated cardiac lymphangiogenesis to improve TAC‐induced cardiac dysfunction. Persistent pressure overload reduces VEGF‐C and VEGFR‐3 expression and inactivates the downstream signals (ATK/ERK, CaNA/NFATc1/FOXC2, and CX43), which inhibits cardiac lymphangiogenesis and increases cardiac edema thereby leading to cardiac hypertrophy, fibrosis, inflammation, apoptosis, and contractile dysfunction in mice. Conversely, stimulation of cardiac lymphangiogenesis by application of VEGF‐C156S prevents these effects, and this represents a new therapeutic pathway for improving cardiac dysfunction after pressure overload.

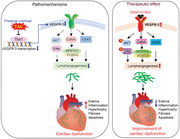

**Abbreviations**
ANF: atrial natriuretic factor
BNP: brain natriuretic peptide
CaNA: calcineurin A
CCL21: C‐C motif chemokine ligand 21
dP/dt_max_: maximal rate of increase in left ventricular pressure
dP/dt_min_: maximal rate of decrease in left ventricular pressure
Ea: arterial elastance (a measure of ventricular afterload)
EF: ejection fraction
FLT‐4: Fms‐like tyrosine kinase receptor 4
FS: fractional shortening
HUVECs: human umbilical vein endothelial cells
I/R: ischemia/reperfusion
LECs: lymphatic endothelial cells
LVSP: left ventricle systolic pressure
LYVE‐1: lymphatic vessel hyaluronan receptor‐1
MI: myocardial infarction
MRI: magnetic resonance imaging
NRCMs: neonatal rat cardiomyocytes
Pdpn: podoplanin
Prox1: prospero‐related homeobox transcription factor 1
PV: pressure‐volume
qPCR: quantitative real‐time PCR
SV: stroke volume
TAC: transverse aortic constriction
Tau: relaxation time constant
VEGF‐C: vascular endothelial growth factor‐C
VEGFR‐3: vascular endothelial growth factor receptor‐3
α‐SMA: α‐smooth muscle actin

## 1 INTRODUCTION

Pathological cardiac hypertrophy is a main risk factor for many serious heart diseases.^1^ The development of cardiac hypertrophy in response to volume or pressure overload is initially considered an adaptive response but may eventually lead to heart failure (HF).^1^, ^2^ Despite the existence of therapies that can improve cardiac dysfunction in patients with HF, the mortality rate remains high, suggesting an urgent need for discovery of novel targets or alternative therapeutic strategies for the prevention of HF. Thus far, multiple molecular pathways that differentially regulate adaptive versus maladaptive hypertrophic remodeling have been identified.^3^ However, previous studies elucidating the mechanisms of cardiac hypertrophy have focused largely on cardiomyocytes (CMs) and the vasculature,^1^, ^4^, ^5^ whereas the contribution of lymphatics to pressure overload‐induced cardiac dysfunction remains poorly defined.

The heart has a complex network of lymphatic vessels that are essential for the maintenance of tissue fluid balance, immune cell trafficking, and cardiac function.^6^, ^7^ Several lymphatic markers, including lymphatic vessel hyaluronan receptor‐1 (LYVE‐1), podoplanin (Pdpn), prospero‐related homeobox transcription factor 1 (Prox1), C‐C motif chemokine ligand 21 (CCL21), and vascular endothelial growth factor (VEGF) receptor‐3 (VEGFR‐3), also known as Fms‐like tyrosine kinase receptor 4 (FLT‐4), have been identified and have been found to be specifically or highly expressed in all lymphatic vessels.^6^, ^7^, ^8^ VEGF family members have been reported to be key mediators of blood vessel growth (angiogenesis) and lymphatic vessel growth (lymphangiogenesis). VEGFR‐3 was the first identified lymphatic‐specific growth factor receptor. VEGF‐C/D and VEGFR‐3 are the main components of the apical signaling pathway for the development and maintenance of lymphatic vessels.^9^ Additionally, VEGF‐C is chemotactic for macrophage migration during various pathological conditions, and VEGFR‐3 is highly expressed in peripheral blood monocytes and tissue macrophages.^10^ Interestingly, loss of VEGF‐C function in mice leads to inhibition of lymphatic vessel growth, and VEGFR‐3 deficient mice display a widespread vascular defects and embryonic death.^11^, ^12^, ^13^, ^14^ VEGF‐C mutation in human patients is associated with autosomal dominant Milroy‐like primary lymphedema.^15^ Importantly, impairment of lymphatic vessel function is involved in various pathological conditions, including lymphedema, chylothorax, inflammation, and tumor metastasis.^6^, ^7^ Recently, several studies have indicated a relationship among cardiac lymphangiogenesis, edema, and contractile dysfunction in animals with myocardial infarction (MI) or ischemia/reperfusion (I/R) injury.^16^, ^17^, ^18^, ^19^, ^20^ Interestingly, stimulation of cardiac lymphangiogenesis improves cardiac lymphatic transport and edema and reduces cardiac inflammation and fibrosis thereby improving left ventricular (LV) function.^16^, ^17^, ^18^ Conversely, transport is impaired, and the other processes are aggravated by inhibition of cardiac lymphangiogenesis.^19^, ^20^ However, the functional role of the VEGF‐C‐VEGFR‐3 axis in pressure overload‐induced cardiac hypertrophy and dysfunction remains unknown.

In this study, using VEGFR‐3 knockdown (VEGFR‐3^f/−^) mice or wild‐type (WT) mice treated with a recombinant human VEGF‐C_156S_ mutant, we examined the potential role of VEGF‐C‐VEGFR‐3 signaling in cardiac hypertrophy and dysfunction induced by transverse aortic constriction (TAC). Our results demonstrated that VEGFR‐3 was essential for cardiac lymphangiogenesis, which played a crucial role in the transition from pressure overload‐induced cardiac hypertrophy to HF in mice. Furthermore, administration of VEGF‐C_156S_ prevented and reversed the development of hypertrophic remodeling in mice subjected to overload, suggesting that selective stimulation of cardiac lymphangiogenesis may be a new therapeutic option for the treatment of HF.

## 2 METHODS

### 2.1 Animals and treatment

WT C57BL/6 and Lyve‐1^EGFP/cre^ mice^21^ (hereafter referred to as Lyve‐1^Cre^ mice) were purchased from The Jackson Laboratory (JAX strain number 012601). VEGFR‐3‐floxed (VEGFR‐3^f/f^) mice were purchased from The European Mouse Mutant Archive (EMMA) (ID: EM:09463). Lyve‐1 promoter drives the expression of Cre recombinase in lymphatic endothelial cells (LECs). To delete the VEGFR‐3 gene in LECs, Lyve‐1^Cre^ mice were mated with VEGFR‐3^f/f^ mice to generate Lyve‐1^cre^VEGFR‐3^f/−^ (referred to VEGFR‐3^f/−^) mice because Lyve‐1^cre^VEGFR‐3^−/−^ was embryonic lethal.^14^ Cardiac hypertrophy and HF were established in male WT C57BL/6 mice, VEGFR‐3^f/−^ mice and their WT (VEGFR‐3^f/f^) littermates at 8–10 weeks old via pressure overload induced by TAC surgery as previously described.^2^

Recombinant VEGF‐C_156S_ (a recombinant mutant form of human VEGF‐C, R&D Systems) was intraperitoneally injected daily into WT mice at a low dose (33 ng/g body weight daily, VEGF‐C‐_L_) and a high dose (100 ng/g body weight daily, VEGF‐C‐_H_) beginning 2 days before TAC. Administration was performed daily for 2 weeks and was then changed to every other day until 6 weeks as previously described.^17^, ^18^ VEGF‐C_156S_ is a recombinant form of human VEGF‐C that selectively binds to and activates VEGFR‐3 present in LECs without affinity for VEGFR‐2 or the vascular permeability‐related activities of native VEGF‐C.^12^, ^22^ For cardiac dysfunction reversal, VEGF‐C_156S_ was injected into the mice for 2 weeks after 4 weeks of TAC. The controls were injected with saline (Supplementary material online). All animal experiments were approved by the Committee on the Ethics of Animal Experiments of Dalian Medical University and conformed to the guidelines of directive 2010/63/EU of the European Parliament on the protection of animals used for scientific purposes.

### 2.2 TAC surgery

Cardiac hypertrophy and HF models were induced with sustained pressure overload via TAC surgery, which was performed as previously described.^2^ Briefly, 8‐ to 10‐week‐old male mice were selected at random and anesthetized with ketamine (0.2 g/kg) and xylazine (0.01 g/kg) by intraperitoneal injection. After adequate exposure of the transverse aorta, a 6‐0 nylon suture was placed between the innominate and left carotid arteries, a 27‐gauge blunt needle was placed at the transverse aorta, and two knots were quickly tied against the needle to produce 65%–70% constriction after prompt removal of the needle. Finally, the skin was sutured using a 4‐0 PROLENE suture. For the sham control mice, all operations were performed with an identical procedure except for ligation. We measured aortic velocity to confirm that the percent occlusion was similar throughout the experiments in all included animals.

### 2.3 Echocardiography, magnetic resonance imaging, and hemodynamics

All mice were anesthetized via intraperitoneal injection of ketamine (0.2 g/kg) and xylazine (0.01 g/kg). Echocardiography at the indicated time points after TAC surgery was performed using a 30 MHz probe as previously described.^23^, ^24^ Magnetic Resonance Imaging (MRI) was performed on anesthetized mice, and the cardiac water content (%) was evaluated by T2‐mapping using a 4.7 T horizontal bore scanner (Bruker).^16^ The in vivo LV function was assessed by invasive pressure‐volume (PV) analysis (supplementary material online).^25^

### 2.4 Histological examinations

Mice were killed with an overdose of isoflurane (>5%) at a flow rate of 1 L/min. LV specimens were fixed in 4% paraformaldehyde and embedded in paraffin. Sections (5 μm) were stained with hematoxylin and eosin and with Masson's trichrome for quantification of cardiac fibrosis using a Masson Trichrome Stain Kit (LEAGENE, DC0032).^23^ Rhodamine‐labeled wheat germ agglutinin (WGA, Vector Laboratories, USA) was used to perform WGA staining to evaluate the CM cross‐sectional area according to its instructions (Vector Laboratories, California, USA). For immunohistochemical staining, LV sections (5 μm) were deparaffinized and subjected to antigen retrieval in a citrate antigen retrieval solution. After blocking nonspecific antigens with 3% BSA, the tissues were incubated with primary antibodies against LYVE‐1 (NOVUS, NBP1‐43411, USA, 1:200), VEGFR‐3 (Abcam, ab27278, UK, 1:200), Pdpn (Abcam, ab11936, UK, 1:200), and α‐smooth muscle actin (α‐SMA, Arigo, ARG66381, Taiwan, 1:200) at 4°C overnight. Biotinylated secondary antibody binding was detected by the DAB detection method. For additional immunohistochemical staining, frozen cardiac sections (8 μm) were dried naturally, fixed with 4% paraformaldehyde, blocked with sheep serum, incubated with primary antibodies against LYVE‐1 (NOVUS, NBP1‐43411, US, 1:200), VEGFR‐3 (Abcam, ab27278, UK, 1:200), Pdpn (Abcam, ab11936, UK, 1:200), CD68 (Abcam, ab201340, UK, 1:200), CD86 (Abcam, ab119857, UK, 1:200), CD206 (Arigo, ARG22456, Taiwan, 1:200), CD31 (Arigo, ARG52748, Taiwan, 1:200), and α‐actinin (Sigma, A7811, DE, 1:200), and detected with fluorescence‐conjugated secondary antibodies (Life‐iLab, 1:200, CN). 4′,6‐Diamidine‐2′‐phenylindole dihydrochloride (DAPI) was used as a nuclear stain. LYVE‐1^+^, VEGFR‐3^+^, or Pdpn^+^ vascular structures (longitudinal or lumenized) were considered lymphatic vessels or capillaries (initial lymphatics). The lymphatic vessels that were identified in the subepicardial and myocardial layers were counted.^26^, ^27^ and are referred to as “cardiac lymphatic vessels or evidence of lymphangiogenesis” throughout the manuscript. The areas of CM surface area, the fibrotic area, and the numbers of lymphatic vessels, CD68^+^ macrophages, and CD31^+^ vascular capillaries were analyzed using a Labophot 2 microscope (Nikon, Tokyo, Japan). Quantitative analysis of the positive areas was performed with ImageJ software. The detailed methods are described in the supplementary material online.^16^

### 2.5 TUNEL assay for CM apoptosis

CM apoptosis detection was performed by using the Dead End Fluorometric TUNEL Assay Apoptosis Detection Kit (US EVERBRIGHT, T6014) according to the manufacturer's instructions. Briefly, frozen heart sections were dried naturally and fixed with 4% paraformaldehyde and then incubated with a TUNEL reaction mixture of terminal deoxynucleotidyl transferase and label solution in a humidified atmosphere for 60 min at 37°C in the dark. The myocardium was stained with α‐actinin antibody (1:400, Sigma) and TRITC‐conjugated secondary antibody to identify myocytes, and the nuclei were counterstained with DAPI. Samples were directly detected under a fluorescence microscope for analysis, and the number of TUNEL‐positive myocyte nuclei was counted at 200x magnification.

### 2.6 Gravimetry

The cardiac water content (%) was quantified by the wet weight‐dry weight method (supplementary material online).^16^

### 2.7 Quantitative real‐time PCR analysis

Total mRNA was extracted from fresh mouse heart samples or cultured cells by the TRIzol reagent method (Sango Biotech, B511311). To obtain cDNA, 1 μg of total mRNA from each group was reverse‐transcribed with a PrimeScript RT Reagent Kit with gDNA Eraser (Yeasen, 11141ES60). Real‐time PCR (qPCR) analysis was performed by using a SYBR Green Premix Pro Taq HS qPCR Kit (Accurate Biotechnology Co., Ltd., Hunan, AG11701) with an Applied Biosystems 7500 Fast instrument (ABI, USA). The mRNA expression levels of atrial natriuretic factor (ANF), brain natriuretic peptide (BNP), collagen I, collagen III, α‐SMA and CD31 were normalized to that of the internal reference gene GAPDH. The primer sequences are described in detail in the supplementary material online, Table S2 and were based on those described previously.^23^

### 2.8 Western blot analysis

Total protein was extracted from fresh tissue or cultured cells by using Tissue Protein Extraction Reagent (Keygenbio, KGP250) containing protease and phosphatase inhibitors according to the manufacturer's instructions and centrifuge tube (Guangzhou Jet Bio‐Filtration Co., Ltd). Western blot analysis was performed as described previously.^23^ The primary antibodies anti‐VEGF‐C (22601‐1‐AP, 1:500), anti‐Bcl‐2 (26593‐1‐AP, 1:1000), and FOXC2 (23066‐1‐AP, 1:1000) were purchased from Proteintech (Wuhan, CN); anti‐VEGFR‐3 (ab27278, 1:1000) and anti‐TBX1 (ab109313, 1:1000) were purchased from Abcam (Cambridge, UK); anti‐phospho‐AKT (9271S, 1:500), anti‐AKT (9272S, 1:1000), anti‐phospho‐ERK1/2 (4370S, 1:500), anti‐ERK1/2 (4695S, 1:1000), anti‐CaNA (2614S, 1:1000), anti‐Bax (2772S, 1:1000), anti‐VEGFR‐2 (9698, 1:500), and anti‐GAPDH (2118S, 1:1000) were obtained from Cell Signaling Technology, Inc (Danvers, MA, USA); anti‐NFATc1 (MA3‐024, 1:1000) was purchased from Invitrogen (Carlsbad, CA, USA); and anti‐connexin 43 (CX43, ARG55217, 1:1000) and anti‐VEGF‐D (ARG58713, 1:1000) were purchased from Arigo (Taiwan, CN). The anti‐rabbit or anti‐mouse horseradish peroxidase‐conjugated secondary antibodies were purchased from Sino Biological Inc. (1:2000).

### 2.9 Statistics

All results are expressed as the mean ± standard deviation (mean ± SD). The statistical analyses were performed with GraphPad Prism 8 software. A normality test (Shapiro‐Wilk) was performed to determine whether the data were normally distributed. If the data were normally distributed, Student's *t* test was used to determine the significant differences between two groups. If the data were not normally distributed, the Mann‐Whitney test was used. One‐way ANOVA was used to analyze the significant differences among multiple groups. If ANOVA demonstrated a significant effect, post hoc pairwise comparisons were made with Fisher's least significant difference test. Values of *p* < 0.05 were considered to indicate statistical significance (supplementary material online).

## 3 RESULTS

### 3.1 Kinetics of cardiac function, hypertrophy, and lymphangiogenesis after pressure overload

**FIGURE 1:**
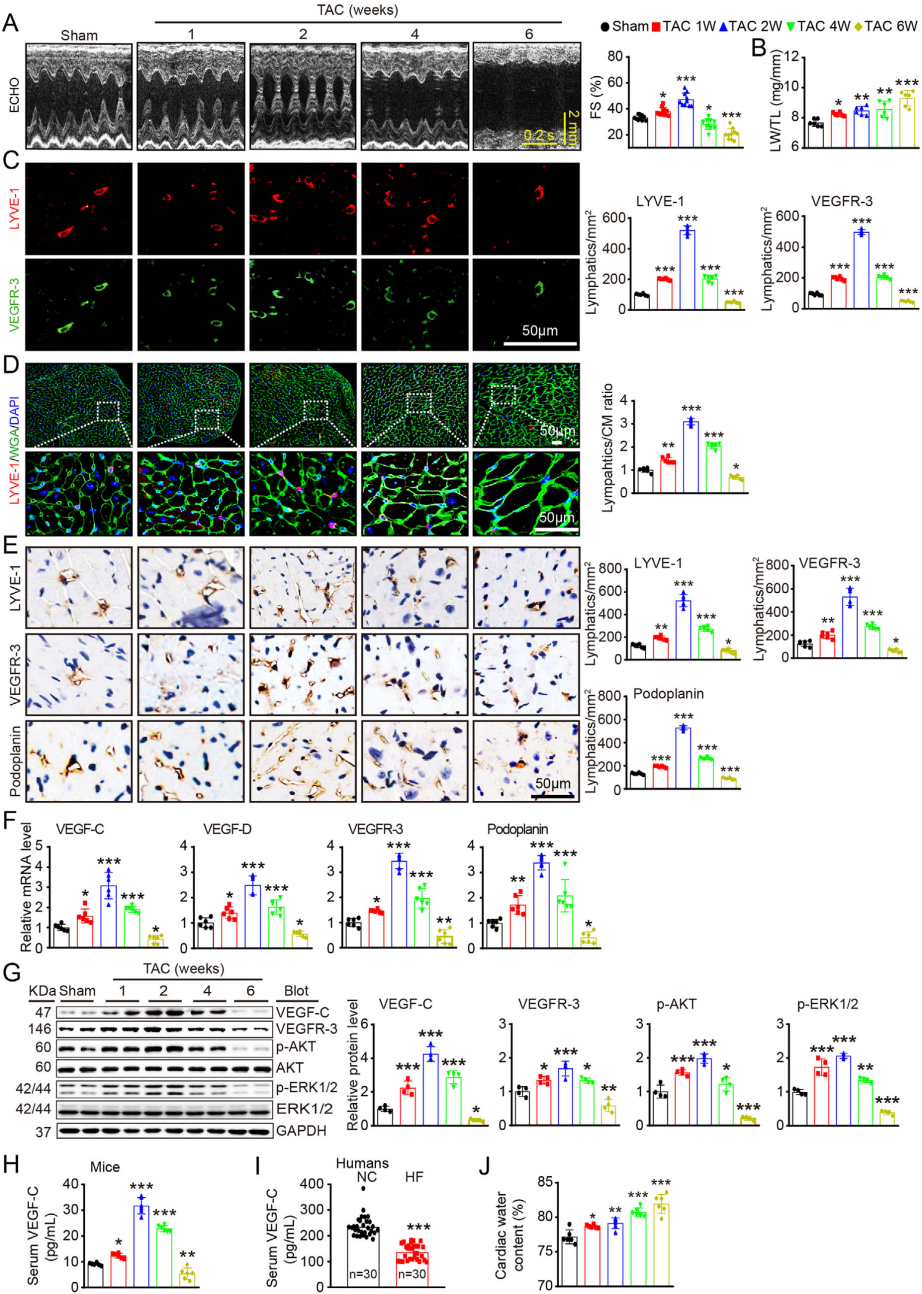
Cardiac function, hypertrophy, and lymphangiogenesis after pressure overload. WT mice were subjected to sham or TAC surgery and remained under sham or TAC conditions for 1–6 weeks. (A) M‐mode echocardiography of the left ventricular (LV) chamber (left) and measurement of LV fraction shortening (FS%) (right, *n* = 10). (B) Lung weight/tibial length (LW/TL) ratio (*n* = 6). (C) Heart sections stained with an antibody against LYVE‐1 (red) or VEGFR‐3 (green) (left, scale bar: 50 μm) and quantification of LYVE‐1^+^ or VEGFR‐3^+^ lymphatic vessels (right, *n* = 6). (D) Heart sections stained with an anti‐LYVE‐1 antibody (red), tetramethylrhodamine isothiocyanate (TRITC)‐labeled wheat germ agglutinin (WGA) (green) and DAPI (blue) (left, scale bar: 50 μm) and the LYVE‐1^+^ vessel to cardiomyocyte (CM) ratio (right, *n* = 6). (E) Immunohistochemical analysis of the lymphatic markers LYVE‐1, VEGFR‐3, and Podoplanin in the heart (left, scale bar: 50 μm) and quantification of the LYVE‐1^+^, VEGFR‐3^+^, and Podoplanin^+^ lymphatic vessels (right, *n* = 6). (F) qPCR analyses of VEGF‐C, VEGF‐D, VEGFR‐3, and Podoplanin mRNA levels (*n* = 6). (G) Immunoblot analysis of the VEGF‐C, VEGFR‐3, p‐AKT, AKT, p‐ERK1/2, and ERK1/2 proteins in the heart (left) and quantification of these proteins (right, *n* = 4). GAPDH was used as an internal control. (H) ELISA assays of the serum VEGF‐C concentrations in mice after sham or TAC surgery. (I) ELISA assays of the serum VEGF‐C concentrations in patients with heart failure (HF, *n* = 30) and normal controls (NCs, *n* = 30). (J) Gravimetric assessment of the cardiac water content (%) in mice as determined by the cardiac dry weight to wet weight ratio (*n* = 6). The data are presented as the mean ± SD, and *n* represents the number of mice or patients per group. Statistical analysis was performed with one‐way ANOVA; *p < 0.05, **p < 0.01, and ***p < 0.001 versus sham or NCs

To determine whether there was a potential relationship between cardiac lymphatic vessels and cardiac hypertrophy and function, we first established a mouse model of hypertrophy with cardiac dysfunction induced by TAC surgery. Echocardiography revealed that cardiac contractile function, as reflected by LV fractional shortening (FS%) (Figure 1A), was enhanced until week 2 and then significantly reduced from weeks 4–6 after TAC with LV chamber dilation, as indicated by decreased LV anterior wall (AW) and posterior wall (PW) thickness and increased LV inner diameter (LVID) (supplementary material online, Table S3). The lung weight/tibial length (LW/TL) ratio was substantially higher in TAC‐treated mice than in untreated mice, indicating pulmonary edema due to cardiac contractile insufficiency (Figure 1B). Moreover, LV and cellular hypertrophy, as indicated by the heart weight/TL (HW) and heart weight/body weight (HW/BW) ratios and the myocyte cross‐sectional area developed in a time‐dependent manner and peaked at week 6 with LV dilation after TAC (supplementary material online, Figures S1A and S1B). Accordingly, compared with the sham control mice, the TAC‐operated mice exhibited time‐dependent increases in myocardial fibrosis and ANF, BNP, α‐SMA, collagen I and collagen III expression (supplementary material online, Figures S1C and S1D). These results suggest that pressure overload initially induced adaptive hypertrophy with preserved cardiac function (weeks 1–2) but that sustained pressure overload resulted in maladaptive hypertrophy and severe contractile dysfunction (weeks 4–6).

Moreover, compared with those in sham control hearts, the number of TUNEL^+^ myocytes and the apoptotic signal Bax/Bcl‐2 ratio in TAC‐operated hearts were increased in a time dependent manner (supplementary material online, Figures S1E and S1F). In addition, the number of CD31^+^ microvessels and the mRNA level of CD31 increased until week 2 and decreased thereafter (supplementary material online, Figures S1G and S1H). There were no significant differences in cardiac function, hypertrophy, and fibrosis between the two time points in the control groups (data not shown).

Cardiac lymphatic vessels are essential for fluid transport and tissue homeostasis.^7^ We first assessed molecular and structural changes related to cardiac lymphangiogenesis by examining lymphatic markers such as LYVE‐1, VEGFR‐3, and Pdpn during the 6 weeks of pressure overload. In agreement with the changes in the severity of cardiac dysfunction after TAC (Figures 1A and 1B), the density of myocardial LYVE‐1^+^ and VEGFR‐3^+^ lymphatic capillaries and the LYVE‐1^+^ lymphatic‐to‐WGA^+^ CM ratio were both markedly increased in week 1, peaked in week 2, and then remarkably decreased in weeks 4–6 (Figures 1C and 1D). The change in lymphatic vessels in the heart was further confirmed by immunohistochemical staining for LYVE‐1, VEGFR‐3, and Pdpn (Figure 1E) and qPCR analysis for the mRNA levels of lymphatic‐specific markers (VEGF‐C, VEGF‐D, VEGFR‐3 and Pdpn) (Figure 1F). The protein levels of VEGF‐C, VEGFR‐3, p‐AKT and p‐ERK1/2 in the heart and the concentration of VEGF‐C in the serum (Figures 1G and 1H) from mice were consistent with alterations in cardiac lymphangiogenesis of during 6 weeks of TAC surgery. In addition, serum VEGF‐C concentrations were lower in human patients with HF than in normal controls (Figure 1I; supplementary material online, Table S1). Finally, we measured cardiac tissue hydration via gravimetry. Compared with the sham control mice, the TAC‐operated mice exhibited time‐dependent increases in cardiac water content (%), as reflected by the wet‐dry weight ratios (Figure 1J), indicating the presence of cardiac edema.

It has been reported that LYVE‐1 is expressed not only in LECs but also in myeloid cells such as macrophages. We then analyzed LYVE‐1^+^ and CD68^+^ cells in the heart at different time points after TAC surgery. Immunostaining revealed that the change in total LYVE‐1^+^ cells was consistent with the number of LYVE‐1^+^/VEGFR‐3^+^ cells in the heart, whereas the number of CD68^+^ macrophages in the heart increased time‐dependently after TAC surgery (supplementary material online, Figure S1I). Notably, the percentage of LYVE‐1^+^CD68^+^ cells was less than 4% at different time points, and the ratio of LYVE‐1^+^CD68^+^ cells to LYVE‐1^+^ cells was less than 2% (supplementary material online, Figure S1I), suggesting that the majority of LYVE‐1^+^ cells in the heart during pressure overload are LECs. Overall, these results indicate that cardiac lymphangiogenesis is increased in the early phase and decreased in the late phase and may be involved in the transition from pressure overload‐induced cardiac hypertrophy to HF.

### 3.2 VEGFR‐3 is required for maintenance of cardiac lymphangiogenesis

**FIGURE 2:**
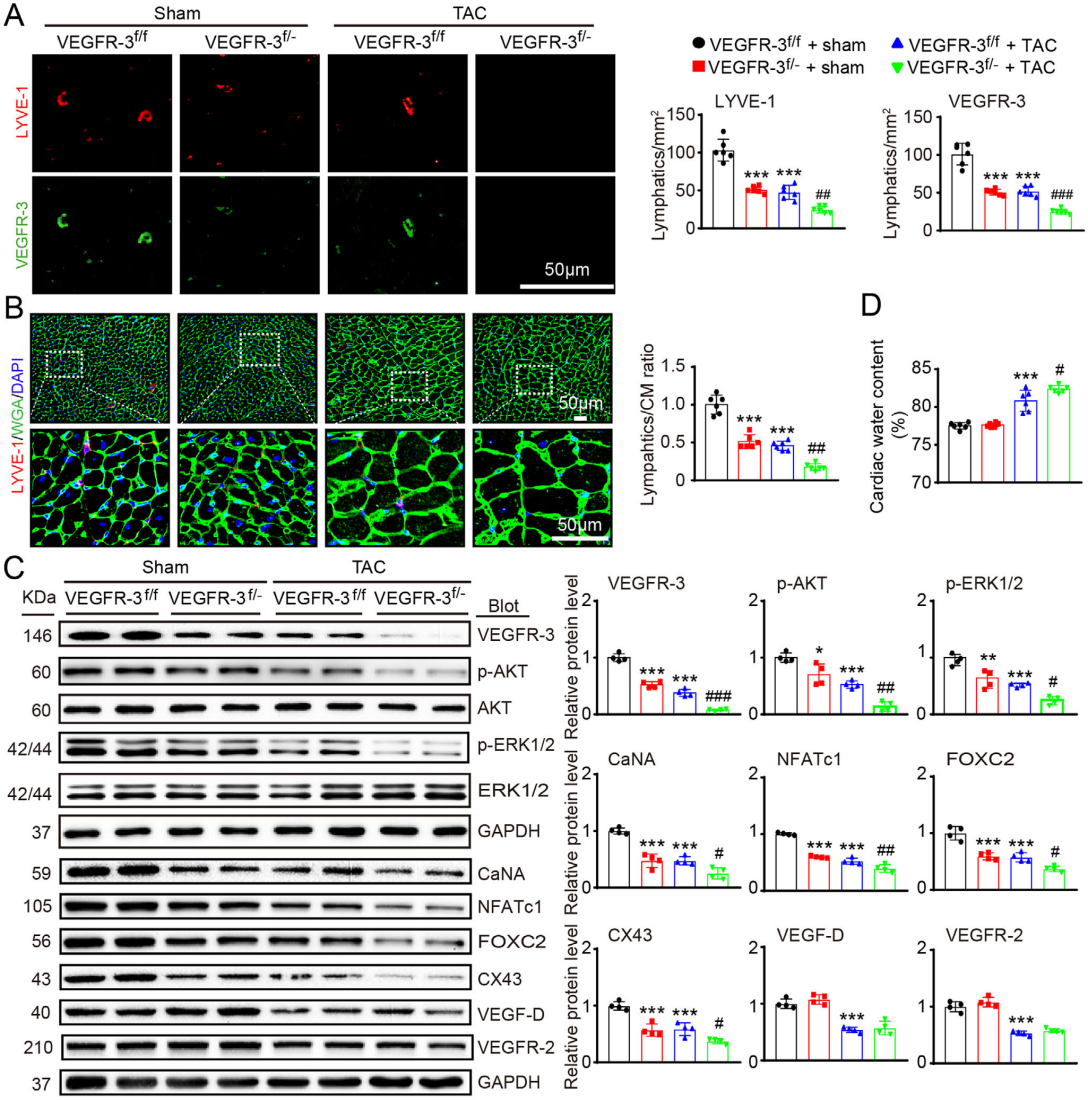
Knockdown of VEGFR‐3 impairs cardiac lymphangiogenesis and causes edema after pressure overload. VEGFR‐3 knockdown (VEGFR‐3^f/−^) mice and their WT (VEGFR‐3^f/f^) littermates were subjected to sham or TAC surgery and remained under sham or TAC conditions for 6 weeks. (A) Heart sections stained with an antibody against LYVE‐1 (red) or VEGFR‐3 (green) (left, scale bar: 50 μm) and quantification of LYVE‐1^+^ or VEGFR‐3^+^ lymphatic vessels (right, *n* = 6). (B) Heart sections stained with an anti‐LYVE‐1 antibody (red), TRITC‐labeled WGA (green) and DAPI (blue) (left, scale bar: 50 μm) and the LYVE‐1^+^ vessel to CM ratio (right, *n* = 6). (C) Immunoblot analysis of the VEGFR‐3, p‐AKT, AKT, p‐ERK1/2, ERK1/2, calcineurin A (CaNA), NFATc1, FOXC2, CX43, VEGF‐D, and VEGFR‐2 proteins in the heart (left) and quantification of these proteins (right, *n* = 4). GAPDH was used as an internal control. (D) Gravimetric assessment of the cardiac water content (%) as determined by the cardiac dry weight‐to‐wet weight ratio (*n* = 6). The data are presented as the mean ± SD, and *n* represents the number of animals per group. Statistical analysis was performed with one‐way ANOVA; *p < 0.05, **p < 0.01, and ***p < 0.001 versus VEGFR‐3^f/f^ + sham; ^#^ *p *< 0.05, ^##^ *p *< 0.01, and ^###^ *p *< 0.001 versus VEGFR‐3^f/f^ + TAC

To ascertain the in vivo role of VEGFR‐3 in cardiac lymphangiogenesis, LYVE‐1 promoter‐specific VEGFR‐3 knockdown (VEGFR‐3^f/−^) mice were generated. VEGFR‐3^f/−^ mice and their WT (VEGFR‐3^f/f^) littermates were subjected to sham, or TAC surgery remained under sham or TAC conditions for 6 weeks. VEGFR‐3^f/f^ mice showed significant reductions in myocardial LYVE‐1^+^ and VEGFR‐3^+^ lymphatic capillaries and the LYVE‐1^+^ lymphatic‐to‐WGA^+^ CM ratio, which was further reduced in VEGFR‐3^f/−^ mice after sham or TAC surgery (Figures 2A and 2B). Consistent with these results, the protein levels of VEGFR‐3 and downstream mediators, including p‐AKT, p‐ERK1/2, calcineurin A (CaNA), NFATc1, FOXC2, and CX43, in VEGFR‐3^f/−^ hearts were markedly lower than those in VEGFR‐3^f/f^ hearts (Figure 2C). However, VEGRR‐3 knockdown did not influence the protein levels of VEGF‐D and VEGFR‐2 after sham or TAC surgery (Figure 2C). Furthermore, gravimetry analysis showed that the total cardiac water content (%) was substantially higher in VEGFR‐3^f/−^ mice than in VEGFR‐3^f/f^ mice after TAC surgery but was similar between the two groups after sham surgery (Figure 2D), suggesting that mice with VEGFR‐3 knockdown can maintain cardiac water balance at baseline. Together, these results indicate that VEGFR‐3 knockdown impairs cardiac lymphangiogenesis, leading to cardiac edema after long‐term pressure overload.

### 3.3 Knockdown of VEGFR‐3 exacerbates cardiac hypertrophy and dysfunction

**FIGURE 3:**
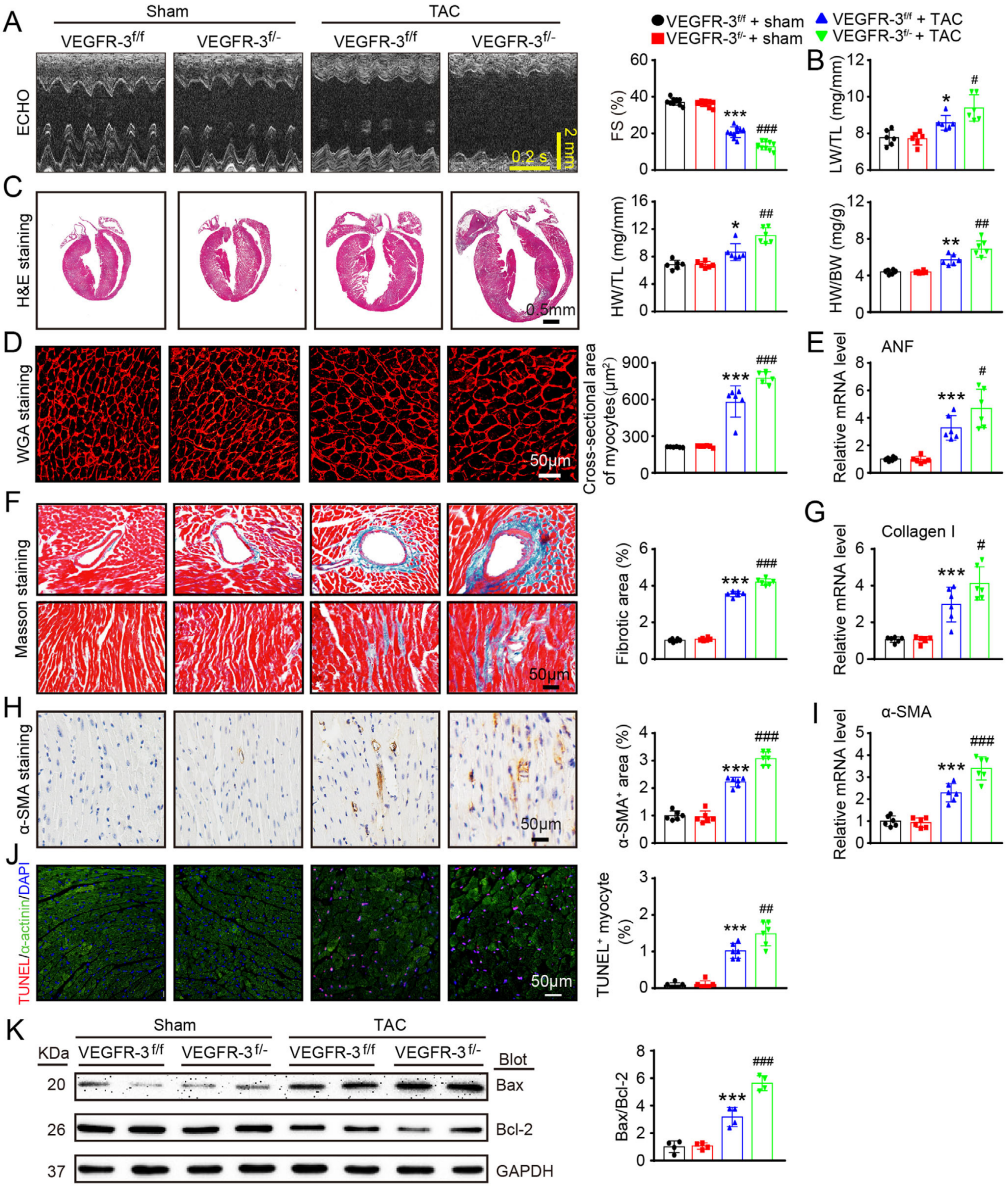
Knockdown of VEGFR‐3 augments cardiac dysfunction, hypertrophy, fibrosis, and apoptosis in mice after pressure overload. VEGFR‐3 knockdown (VEGFR‐3^f/−^) mice and their WT (VEGFR‐3^f/f^) littermates were subjected to sham or TAC surgery and remained under sham or TAC conditions for 6 weeks. (A) M‐mode echocardiography of the LV chamber (left) and measurement of LV FS% (right, *n* = 10). (B) LW/TL ratio (*n* = 6). (C) H&E staining of heart sections (left, scale bar: 0.50 mm) and HW/BW and HW/TL ratios (right, *n* = 6). (D) TRITC‐labeled WGA staining of heart sections (left, scale bar: 50 μm) and quantification of the myocyte cross‐sectional area (right, *n* = 6, 200 cells counted per heart). (E) qPCR analyses of ANF mRNA levels (*n* = 6). (F) Masson's trichrome staining of heart sections (left) and quantification of the fibrotic area (right, *n* = 6). Scale bar: 50 μm. (G) qPCR analyses of collagen I mRNA level (*n* = 6). (H) Immunohistochemical staining with an anti‐α‐SMA antibody (left) and quantification of the α‐SMA^+^ area (right, *n* = 6). Scale bar: 50 μm. (I) qPCR analyses of α‐SMA mRNA levels (*n* = 6). (J) Heart sections stained with TUNEL (red), α‐actinin (green), and DAPI (blue) (left, scale bar: 50 μm) and quantification of TUNEL^+^ myocytes (right, *n* = 6). (K) Immunoblot analysis of Bax and Bcl‐2 protein levels in the heart (left) and quantification of the Bax to Bcl‐2 ratio (right, *n* = 4). GAPDH was used as an internal control. The data are presented as the mean ± SD, and *n* represents the number of animals per group. Statistical analysis was performed with one‐way ANOVA; *p < 0.05, **p < 0.01, and ***p < 0.001 versus VEGFR‐3^f/f^ + sham; ^#^ *p *< 0.05, ^##^ *p *< 0.01, and ^###^ *p *< 0.001 versus VEGFR‐3^f/f^ + TAC

We then assessed whether VEGFR‐3 is involved in regulating cardiac hypertrophy and function in vivo. Following 6 weeks of TAC surgery, VEGFR‐3^f/f^ mice showed characteristics of HF, as indicated by significant decreases in FS%, left ventricular anterior wall (LVAW) and left ventricular posterior wall (LVPW) thickness and an increase in LVID, which was further aggravated in VEGFR‐3^f/−^ mice (Figure 3A, supplementary material online, Table S4). A marked increase in the LW/TL ratio in TAC‐subjected VEGFR‐3^f/−^ mice further confirmed cardiac contractile insufficiency (Figure 3B). Cardiac hypertrophy, as reflected by increases in heart size, HW/BW and HW/TL ratios, myocyte cross‐sectional area, and ANF mRNA expression, became even more prominent in VEGFR‐3^f/−^ mice than in VEGFR‐3^f/f^ mice after TAC stress (Figures 3C‐3E). LV fibrosis, as indicated by enhancement of the perivascular and interstitial fibrotic area, the number of α‐SMA^+^ myofibroblasts and the mRNA expression of collagen I and α‐SMA, was more visible in VEGFR‐3^f/−^ mice than in VEGFR‐3^f/f^ mice (Figures 3F‐3I). Consistently, the number of TUNEL^+^ CM and the Bax/Bcl‐2 ratio were also higher in TAC‐subjected VEGFR‐3^f/−^ mice than in TAC‐subjected VEGFR‐3^f/f^ mice (Figures 3J and 3K). There were no differences in these variables between VEGFR‐3^f/−^ mice and VEGFR‐3^f/f^ controls after sham surgery (Figures 3A‐3K, supplementary material online, Table S4), indicating that VEGFR‐3 knockdown has no influence on cardiac remodeling at baseline. These results demonstrate that VEGFR‐3 knockdown mice are more vulnerable than WT mice to prolonged pressure overload‐induced HF.

We also determined the effect of VEGFR‐3 knockdown on the polarization of newly recruited macrophages in the heart after 2 weeks of TAC. Immunoblot analysis showed that the expression of VEGFR‐3 protein was markedly lower in VEGFR‐3^f/‐^ macrophages than in VEGFR‐3^f/f^ cells (supplementary material online, Figure S2A). Moreover, the percentages of total CD68^+^ macrophages and CD68^+^CD86^+^ M1 macrophages as well as the mRNA levels of M1 markers (IL‐1β, IL‐6, TNF‐α, and MCP‐1) were higher, whereas the percentage of CD68^+^CD206^+^ M2 macrophages and the mRNA levels of M2 markers (Arg1, Ym1, and IL‐10) were lower in VEGFR‐3^f/−^ mice than in VEGFR‐3^f/f^ mice (supplementary material online, Figures S2B‐S2E). ELISA assays further confirmed that the levels of circulating pro‐inflammatory cytokines (IL‐1β, IL‐6, TNF‐α, and MCP‐1) were higher in VEGFR‐3^f/−^ mice than in VEGFR‐3^f/f^ mice (supplementary material online, Figure S2F). In addition, the protein levels of VEGFR‐3 in the lungs, liver, and intestines were lower in VEGFR‐3^f/−^ mice than in VEGFR‐3^f/f^ mice (supplementary material online, Figure S2G). However, compared with VEGFR‐3^f/f^ controls, VEGFR‐3^f/−^ mice did not exhibit significantly alterations in circulating total cholesterol and triglyceride levels, body weight curves, or systolic blood pressure (supplementary material online, Figures S2H‐S2J). Overall, these findings suggest that VEGFR‐3 knockdown promotes the recruitment and M1 polarization of macrophages, which may contribute to cardiac hypertrophy and dysfunction post‐TAC surgery.

### 3.4 Systemic administration of VEGF‐C_156S_ stimulates myocardial lymphangiogenesis and reduces cardiac edema

**FIGURE 4:**
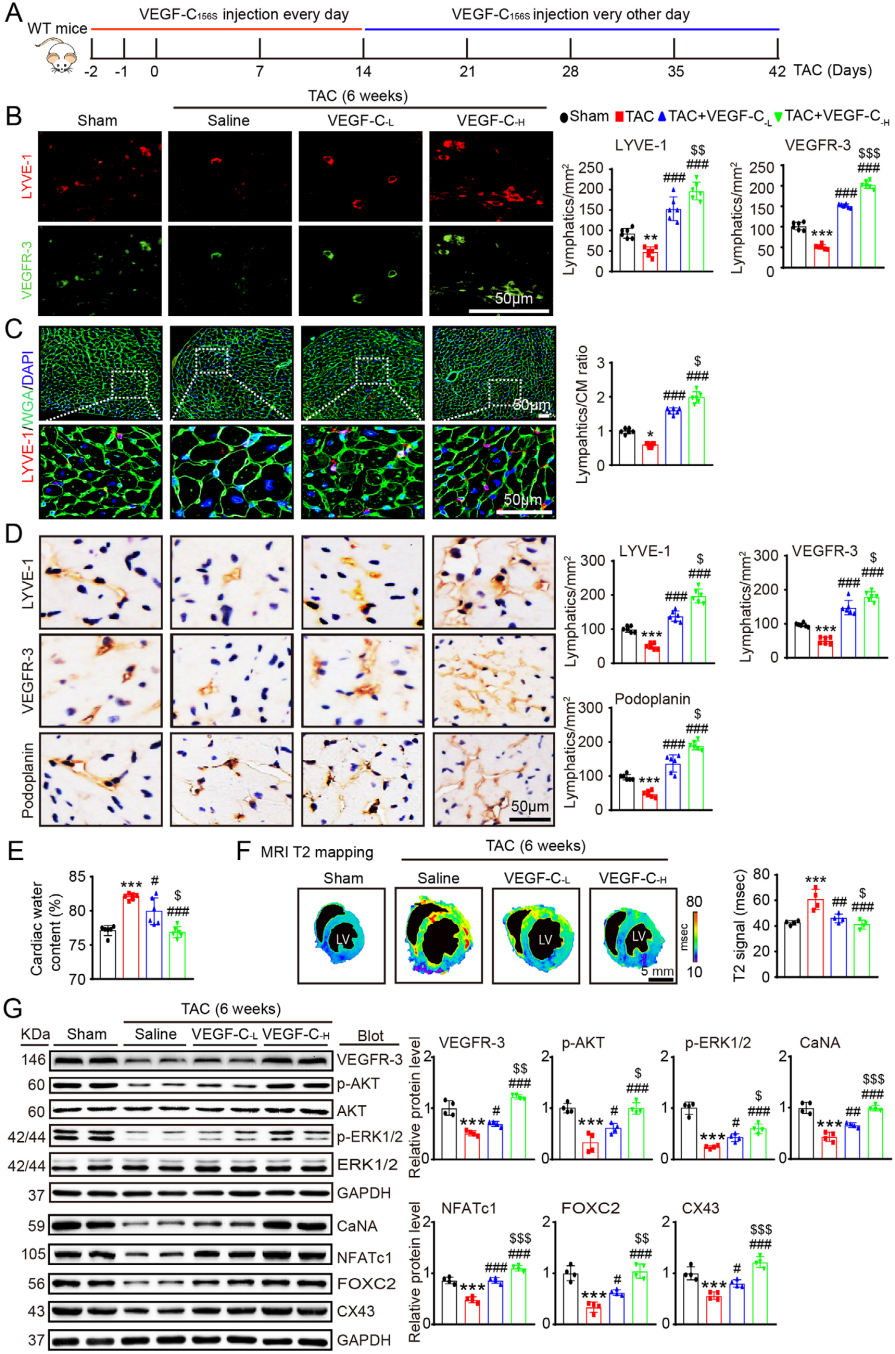
Administration of VEGF‐C_156S_ improves cardiac lymphangiogenesis and edema after pressure overload. (A) Schematic of the methods for treatment of WT mice with saline or VEGF‐C_156S_ at doses of 33 (VEGF‐C_‐L_) and 100 (VEGF‐C_‐H_) ng/g and TAC for 6 weeks. (B) Heart sections stained with an anti‐LYVE‐1 (red) or anti‐VEGFR‐3 antibody (green) (left, scale bar: 50 μm) and quantification of LYVE‐1^+^ or VEGFR‐3^+^ lymphatics (right, *n* = 6). (C) Heart sections stained with an anti‐LYVE‐1 antibody (red), TRITC‐labeled WGA (green) and DAPI (blue) (left, scale bar: 50 μm) and the LYVE‐1^+^ vessel to CM ratio (right, *n* = 6). (D) Immunohistochemical staining of heart sections with an antibody against LYVE‐1, VEGFR‐3 or Podoplanin (left, scale bar: 50 μm) and quantification of LYVE‐1^+^, VEGFR‐3^+^, and Podoplanin^+^ lymphatic vessels (right, *n* = 6). (E) Gravimetric assessment of cardiac water content (%) as determined by the cardiac dry weight to wet weight (*n* = 6). (F) Representative color‐coded images of cardiac MRI T2 mapping in which the turquoise/blue areas are normal tissues and the red/yellow areas exhibit cardiac edema (left) and MRI‐based quantification of cardiac water content (T2 map signal intensity, msec) (right, *n* = 4). (G) Immunoblot analysis of the VEGFR‐3, p‐AKT, AKT, p‐ERK1/2, ERK1/2, CaNA, NFATc1, FOX2C, and CX43 proteins in the heart (left) and quantification of these proteins (right, *n* = 4). GAPDH was used as an internal control. The data are presented as the mean ± SD, and *n* represents the number of animals per group. Statistical analysis was performed with one‐way ANOVA; *p < 0.05, **p < 0.01, and ***p < 0.001 versus sham; ^#^ *p *< 0.05, ^##^ *p *< 0.01, and ^###^ *p *< 0.001 versus TAC + saline; ^$^ *p *< 0.05, ^$$^ *p *< 0.01, and ^$$$^ *p *< 0.001 versus TAC + VEGF‐C_‐L_

To test whether cardiac lymphangiogenesis is a potential therapeutic target for the treatment of HF, WT mice were i.p. administered recombinant human VEGF‐C_156S_ at a dose of 33 (VEGF‐C_‐L_) or 100 (VEGF‐C_‐H_) ng/g and then subjected to TAC for an additional 6 weeks (Figure 4A). By 20 min after injection, the serum human VEGF‐C concentrations in the VEGF‐C‐_L_ and VEGF‐C‐_H_ injected mice had increased to 16.86 ± 2.78 and 33.36 ± 9.72 pg/ml, respectively, as evaluated with a human ELISA kit. Moreover, compared with the saline control mice, the VEGF‐C_156S_‐injected mice exhibited dose‐dependent increases in myocardial LYVE‐1^+^ and VEGFR‐3^+^ lymphatic vessels and the LYVE‐1^+^ lymphatic to WGA^+^ CM ratio 6 weeks after TAC surgery (Figures 4B and 4C). The enhancement of LYVE‐1^+^, VEGFR‐3^+^, and Pdpn^+^ lymphatic vessels in VEGF‐C_156S_‐treated hearts was further confirmed by immunohistochemical staining (Figure 4D). Accordingly, there was a significant reduction of total cardiac water content (%) as assessed by gravimetry assay (Figure 4E) and MRI T2 mapping (Figure 4F) in VEGF‐C_156S_‐treated mice as compared with saline controls, indicating that cardiac lymphatic function had improved. Conversely, the VEGF‐C_156S_‐induced increase in lymphangiogenesis (as indicated by LYVE‐1^+^ and VEGFR‐3^+^ lymphatics and the LYVE‐1^+^ lymphatic to WGA^+^ CM ratio) and reduction in cardiac water content (%) were markedly attenuated in VEGFR‐3^f/−^ mice (supplementary material online, Figures S3A‐S3C). Moreover, compared with saline treatment, VEGF‐C_156S_ treatment resulted in dose‐dependent increases in the protein levels of VEGFR‐3, p‐AKT, p‐ERK1/2, CaNA, NFATc1, FOXC2, and CX43 after TAC surgery (Figure 4G). These results indicate that VEGF‐C_156S_ administration promotes cardiac lymphangiogenesis through VEGFR‐3‐mediated signaling pathways.

**FIGURE 5:**
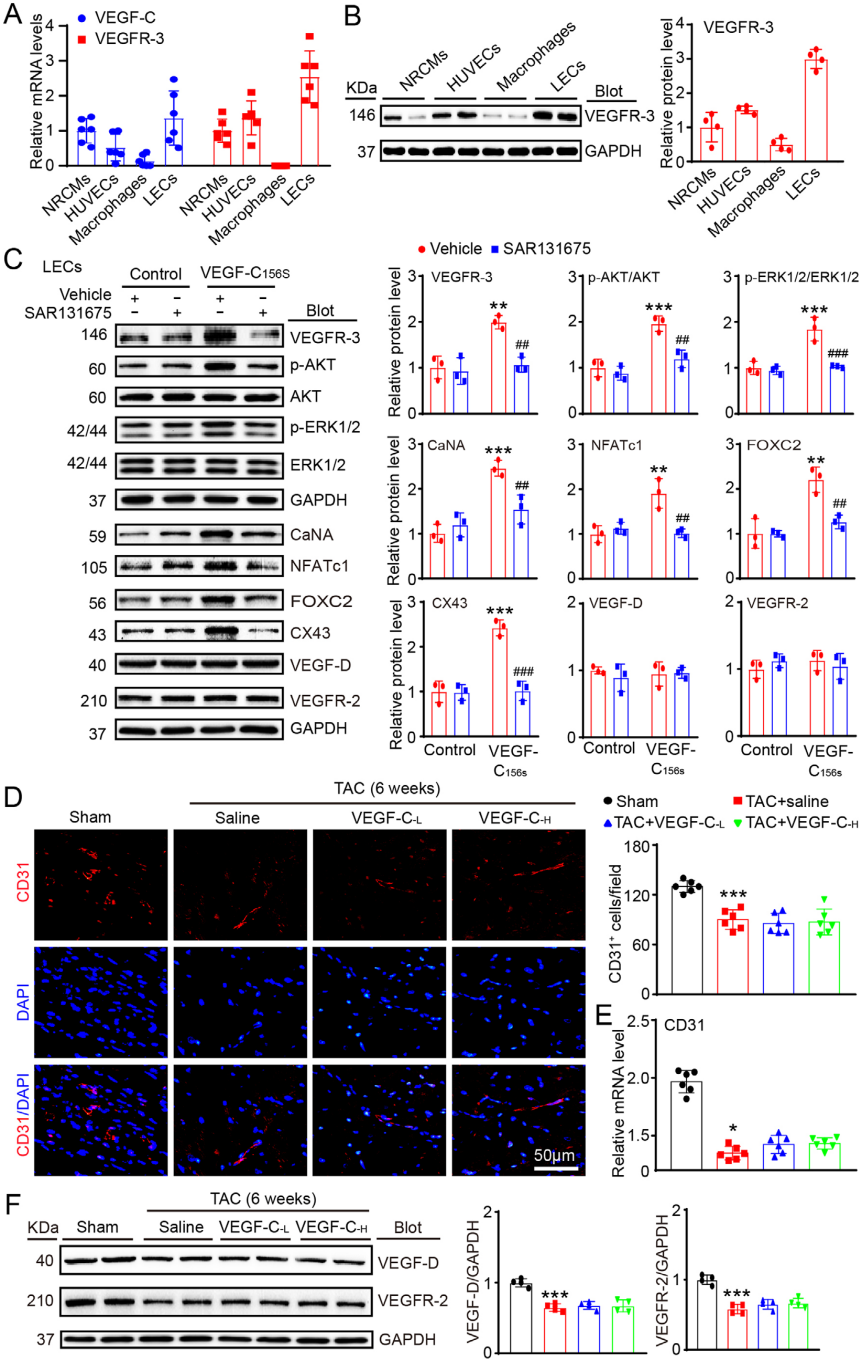
VEGF‐C_156S_ treatment selectively activated VEGFR‐3 signaling in mouse LECs and did not affect cardiac angiogenesis after TAC. (A) qPCR analysis of VEGF‐C and VEGFR‐3 mRNA levels in neonatal rat cardiomyocytes (NRCMs), human umbilical vein endothelial cells (HUVECs), mouse primary macrophages, and mouse LECs. (B) Immunoblot analysis of the VEGFR‐3 protein (left) in different cell types and quantification of this protein (right, *n* = 4). (C) LECs were treated with or without the VEGFR‐3 inhibitor SAR131675 (20 nM) for 30 min and then stimulated with VEGF‐C_156S_ (100 ng/mL) for 24 h. Immunoblot analysis of the VEGFR‐3, p‐AKT, AKT, p‐ERK1/2, ERK1/2, CaNA, NFATc1, FOX2C, CX43, VEGF‐D, and VEGFR‐2 proteins (left) and quantification of these proteins (right, *n* = 3). GAPDH was used as an internal control. (D) WT mice were treated with saline or VEGF‐C_156S_ at doses of 33 (VEGF‐C_‐L_) and 100 (VEGF‐C_‐H_) ng/g daily and subjected to TAC for 6 weeks. Heart sections stained with an anti‐CD31 antibody (red) and DAPI (blue) (left) and quantification of CD31^+^ vessels (right, *n* = 6). Scale bar: 50 μm. (E) qPCR analyses of CD31 mRNA levels (*n* = 6). (F) Immunoblot analysis of the VEGF‐D and VEGFR‐2 proteins and quantification of these proteins (*n* = 4). The data are presented as the mean ± SD, and *n* represents the number of animals per group. Statistical analysis was performed with one‐way ANOVA; *p < 0.05, **p < 0.01, and ***p < 0.001 versus control or sham; ^##^ *p *< 0.01 and ^###^ *p *< 0.001 versus VEGF‐C_156S_ + vehicle

To test whether the effect of VEGF‐C_156S_ on this signaling is specific, we examined VEGFR‐3 expression in different cardiac cell types. We found that the mRNA expression of VEGF‐C and VEGFR‐3 was the most significantly upregulated in mouse LECs and was high in neonatal rat CMs (CMs) and human umbilical vein endothelial cells, but less detectable in bone marrow‐derived macrophages under basal condition, indicating that LECs are the main source of VEGF‐C (Figure 5A). The protein level of VEGFR‐3 in different cardiac cell types was confirmed by immunoblotting analysis (Figure 5B). Furthermore, compared with the control treatment, VEGF‐C_156S_ treatment significantly upregulated the protein levels of VEGFR‐3, p‐AKT, p‐ERK1/2, CaNA, NFATc1, FOXC2, and CX43 except VEGF‐D and VEGR‐2, and this effect was dramatically attenuated by the VEGFR‐3 inhibitor SAR131675 in LECs (Figure 5C). Interestingly, VEGF‐C_156S_ injection after TAC surgery did not significantly affect cardiac angiogenesis in mice, as indicated by the CD31^+^ blood vessels numbers, CD31 mRNA levels, and VEGFR‐2 and VEGF‐D protein levels (Figures 5D‐5F), supporting the idea that VEGF‐C_156S_ does not exert an angiogenic effect^12^, ^28^ and selectively activates VEGFR‐3 signaling in LECs. Collectively, these data indicate that VEGF‐C_156S_ can specifically stimulate lymphangiogenesis by activating VEGFR‐3 signaling in the heart.

### 3.5 Administration of VEGF‐C_156S_ ameliorates cardiac hypertrophy and dysfunction

**FIGURE 6:**
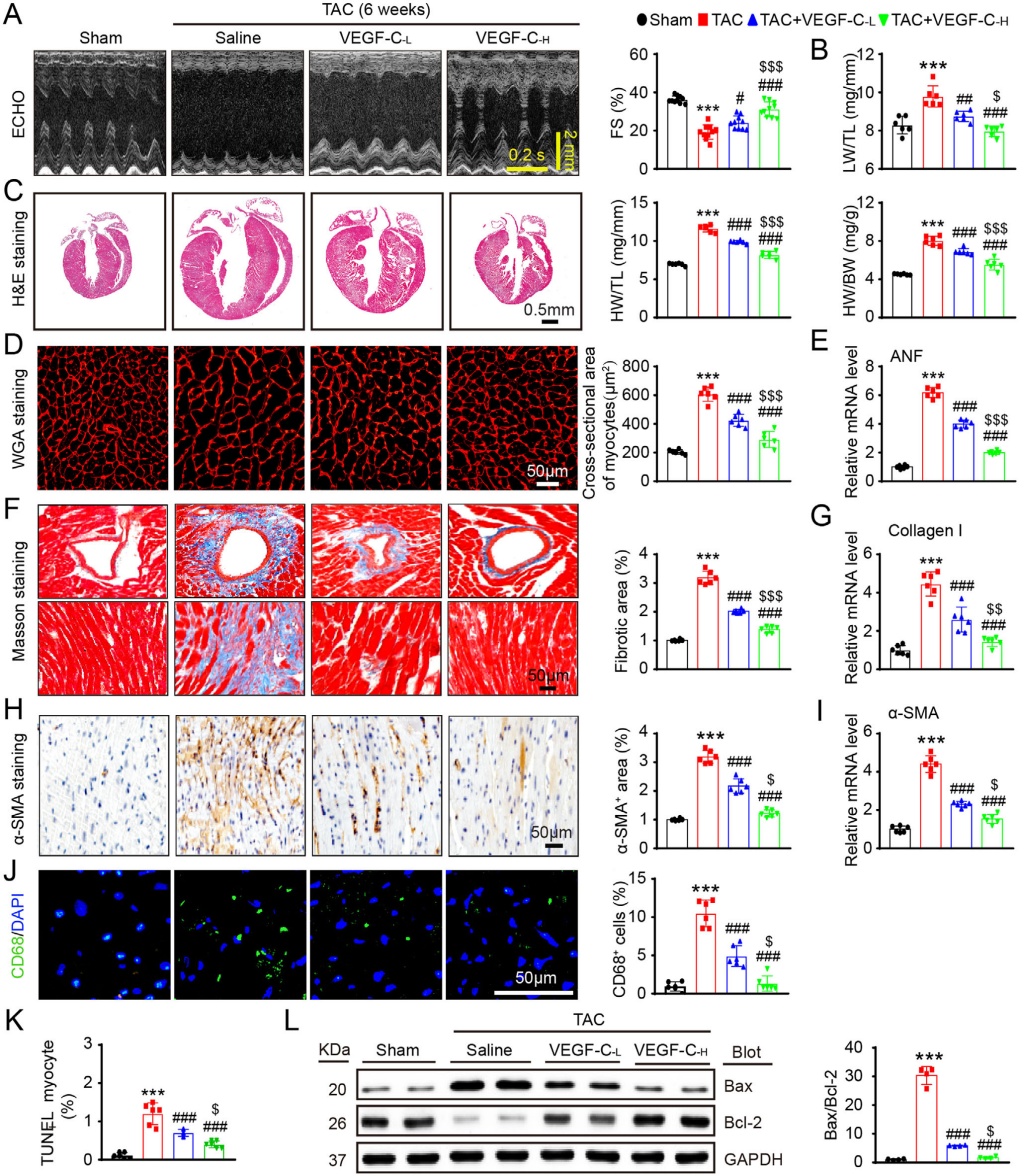
Administration of VEGF‐C_156S_ alleviates pressure overload‐induced cardiac hypertrophy, fibrosis, dysfunction, macrophage infiltration, and apoptosis. WT mice were treated with saline or VEGF‐C_156S_ at doses of 33 (VEGF‐C_‐L_) and 100 (VEGF‐C_‐H_) ng/g and TAC for 6 weeks. (A) M‐mode echocardiography of the LV chamber (left). Measurement of LV FS% (right, *n* = 10). (B) LW/TL ratio (*n* = 6). (C) H&E staining of heart sections (left, scale bar: 0.50 mm) and HW/BW and HW/TL ratios (right, *n* = 6). (D) TRITC‐labeled WGA staining of heart sections (left, scale bar: 50 μm) and quantification of the myocyte cross‐sectional area (right, *n* = 6, 200 cells counted per heart). (E) qPCR analyses of ANF mRNA level (*n* = 6). (F) Masson's trichrome staining of heart sections (left) and quantification of the fibrotic area (right, *n* = 6). Scale bar: 50 μm. (G) qPCR analyses of collagen I mRNA level (*n* = 6). (H) Immunohistochemical staining of heart sections with an anti‐α‐SMA antibody (left) and quantification of the α‐SMA^+^ area (right, *n* = 6). Scale bar: 50 μm. (I) qPCR analyses of α‐SMA mRNA level (*n* = 6). (J) Heart sections were immunostained with an anti‐CD68 antibody (green) and DAPI (blue) (left, scale bar: 50 μm) and quantification of CD68^+^ macrophages (right, *n* = 6). (K) The percentage of TUNEL^+^ cardiomyocytes in the heart (*n* = 6). (L) Immunoblot analysis of Bax and Bcl‐2 protein levels in the heart (left) and quantification of the Bax to Bcl‐2 ratio (right, *n* = 4). GAPDH was used as an internal control. The data are presented as the mean ± SD, *n* represents the number of animals per group. Statistical analysis was performed with one‐way ANOVA; **p < 0.01 and ***p < 0.001 versus sham; ^#^ *p *< 0.05, ^##^ *p *< 0.01, and ^###^ *p *< 0.001 versus TAC + saline; ^$^ *p *< 0.05 versus TAC +VEGF‐C_‐L_

**FIGURE 7:**
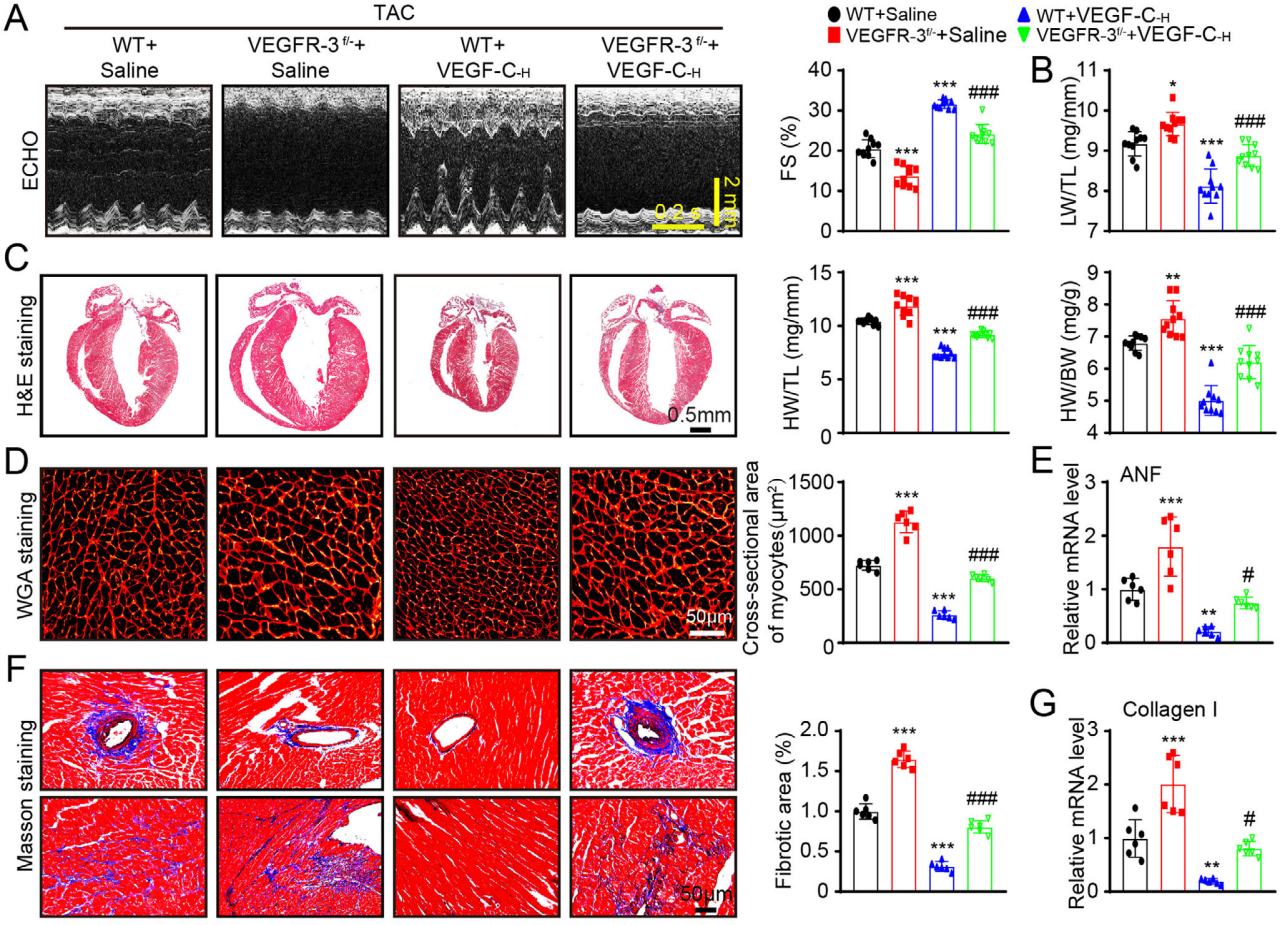
VEGFR‐3 knockdown abrogates the VEGF‐C_156S_‐mediated cardioprotective effect in mice. WT and VEGFR‐3^f/−^ mice were treated with saline or VEGF‐C_156S_ and subjected to TAC for 6 weeks. (A) M‐mode echocardiography of the LV chamber (left) and measurement of LV FS% (right, *n* = 10). (B) LW/TL ratio (*n* = 10). (C) H&E staining of heart sections. Scale bar: 0.50 mm (left) and the HW/BW and HW/TL ratios (right, *n* = 10). (D) TRITC‐labeled WGA staining of heart sections (left, scale bar: 50 μm) and quantification of the myocyte cross‐sectional area (right, *n* = 6, 200 cells counted per heart). (E) qPCR analyses of ANF mRNA level (*n* = 6). (F) Masson's trichrome staining of heart sections (left) and quantification of the fibrotic area (right, *n* = 6). Scale bar: 50 μm. (G) qPCR analyses of collagen I mRNA level (*n* = 6). The data are presented as the mean ± SD, and *n* represents the number of animals per group. Statistical analysis was performed with one‐way ANOVA; *p < 0.05, **p < 0.01, and ***p < 0.001 versus WT + saline; ^#^ *p *< 0.05 and ^###^ *p *< 0.001 versus WT + VEGF‐C‐_H_

We next evaluated the effects of VEGF‐C_156S_ on TAC‐induced cardiac remodeling and dysfunction. Echocardiography showed that TAC‐operated mice had characteristics of HF, including significantly reduced FS% values, LV chamber dilation (decreased LVAW and LVPW thickness), and increased LW/TL ratios, whereas these effects were markedly and dose‐dependently attenuated by administration of VEGF‐C_156S_ (Figures 6A and 6B, supplementary material online, Table S5). Moreover, compared with the saline control treatment, VEGF‐C_156S_ treatment dose‐dependently attenuated cardiac hypertrophy (as indicated by increases in heart size, HW/BW, and HW/TL ratios, myocyte cross‐sectional areas and ANF, mRNA level) and fibrotic responses (as indicated by increases in perivascular and interstitial fibrotic areas, the number of α‐SMA^+^ myofibroblasts and the mRNA expression of collagen I and α‐SMA) after 6 weeks of TAC (Figures 6C‐6I). Moreover, the infiltration of CD68^+^ macrophages, the number of TUNEL^+^ myocytes, and the Bax/Bcl‐2 ratio were also markedly lower in the hearts of VEGF‐C_156S_‐injected mice than in those of saline‐injected control mice after TAC (Figures 6J‐6L), indicating a protective effect against cardiac inflammation and apoptosis. Conversely, VEGF‐C_156S_‐mediated cardioprotective effects, as reflected by improved cardiac dysfunction (FS%), hypertrophy (heart size, HW/BW and HW/TL ratios, myocyte cross‐sectional areas and ANF expression) and fibrosis (fibrotic area and collagen I expression), were all markedly reversed in VEGFR‐3^f/−^ mice (Figures 7A‐7G).

Moreover, we tested the effect of VEGF‐C_156S_ on CM growth in vitro and found that VEGF‐C_156S_ treatment did not increase CM size and the mRNA levels of ANF and BNP compared with control in the presence or absence of SAR131675 (supplementary material online, Figures S4A and S4B), suggesting that VEGF‐C_156S_ has no direct effect on CM hypertrophy. In addition, there were no adverse side effects in mice systemically administered VEGF‐C_156S_. These findings demonstrate that therapeutic stimulation of cardiac lymphangiogenesis with VEGF‐C_156S_ is very effective in preserving cardiac function and preventing hypertrophy development.

**FIGURE 8:**
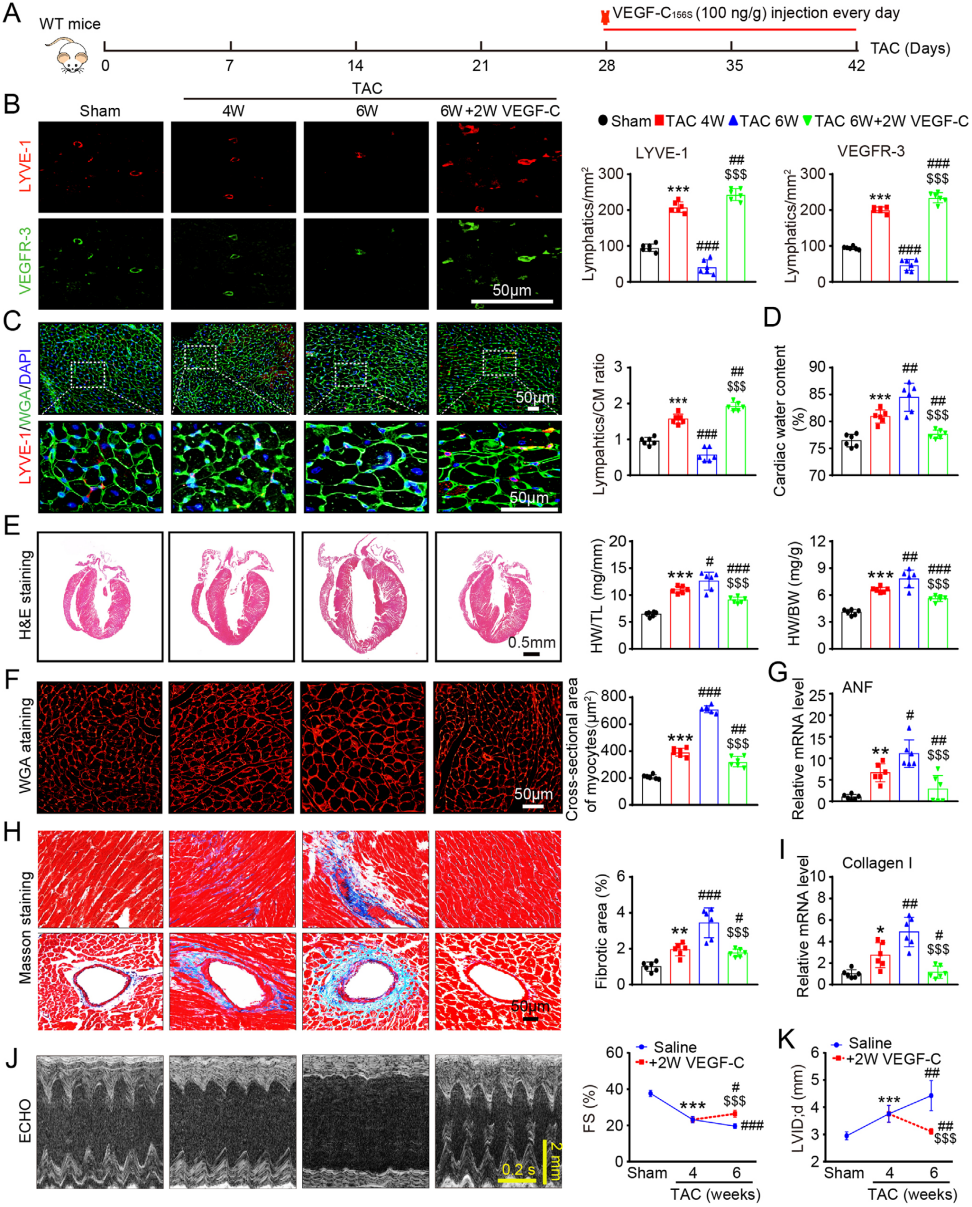
VEGF‐C_156S_ reverses established cardiac hypertrophy and dysfunction. (A) Schematic of the methods for treatment of WT mice that were subjected to TAC for 4 weeks and then administered saline or VEGF‐C_156S_ (100 ng/g daily per animal) and kept under TAC conditions for an additional 2 weeks. (B) Heart sections stained with an anti‐LYVE‐1 (red) or anti‐VEGFR‐3 antibody (green) (left, scale bar: 50 μm) and quantification of LYVE‐1^+^ or VEGFR‐3^+^ lymphatics (right, n = 6). (C) Heart sections stained with an anti‐LYVE‐1 antibody (red), TRITC‐labeled WGA (green), and DAPI (blue) (left, scale bar: 50 μm) and the LYVE‐1^+^ vessel to CM ratio (right, *n* = 6). (D) Gravimetric measurement of cardiac water content (%) (*n* = 6). (E) H&E staining of heart sections (left, scale bar: 0.5 mm) and HW/BW and HW/TL ratios (right, *n* = 6). (F) Representative images of TRITC‐labeled WGA staining of CMs (left, scale bar: 50 μm) and quantification of the myocyte cross‐sectional area (right, *n* = 6, 200 cells counted per heart). (G) qPCR analyses of ANF mRNA level (*n* = 6). (H) Masson's trichrome staining of heart sections (left) and quantification of the fibrotic area (right, *n* = 6). Scale bar: 50 μm. (I) qPCR analyses of collagen I mRNA level (*n* = 6). (J) Echocardiographic assessment of the LV chamber (left) and FS% (right, *n* = 10). (K) Measurement of the LVID (*n* = 10). The data are presented as the mean ± SD, and *n* represents the number of animals per group. Statistical analysis was performed with one‐way ANOVA; *p < 0.05, **p < 0.01, and ***p < 0.001 versus sham; ^#^ *p *< 0.05, ^##^ *p *< 0.01, and ^###^ *p *< 0.001 versus TAC 4 week; ^$$$^ *p *< 0.001 versus TAC 6 week

To further determine potential clinical applications, we tested whether VEGF‐C_156S_ is able to reverse existing cardiac hypertrophy and dysfunction. WT mice were subjected to TAC for 4 weeks, which impaired cardiac lymphangiogenesis; increased the cardiac water content (%), hypertrophy, fibrosis, and LVID; and reduced FS% (Figures 8A‐8K). These mice were then randomly assigned to two groups: one group received VEGF‐C_156S_ (100 ng/g daily per mouse) via intraperitoneal injection for an additional 2 weeks and the other group received only saline (Figure 8A). Compared with the sham control condition, TAC for 6 weeks led to reduced cardiac lymphangiogenesis and severe cardiac edema, which were markedly ameliorated in VEGF‐C_156S_‐treated animals (Figures 8B‐8D). Moreover, the TAC‐induced increases in cardiac hypertrophy, fibrosis, and the mRNA levels of ANF and collagen I in saline‐treated mice were prominently reversed with VEGF‐C_156S_ treatment (Figures 8E‐8I). Importantly, serial echocardiography showed improved contractile function (FS%) and a gradual decline in LVID in VEGF‐C_156S_‐treated mice (Figures 8J and 8K). The other echocardiographic parameters are shown in supplementary material online, Table S6. These results indicate that VEGF‐C_156S_ treatment can effectively alleviate hypertrophy and reverse the progression of HF.

**FIGURE 9:**
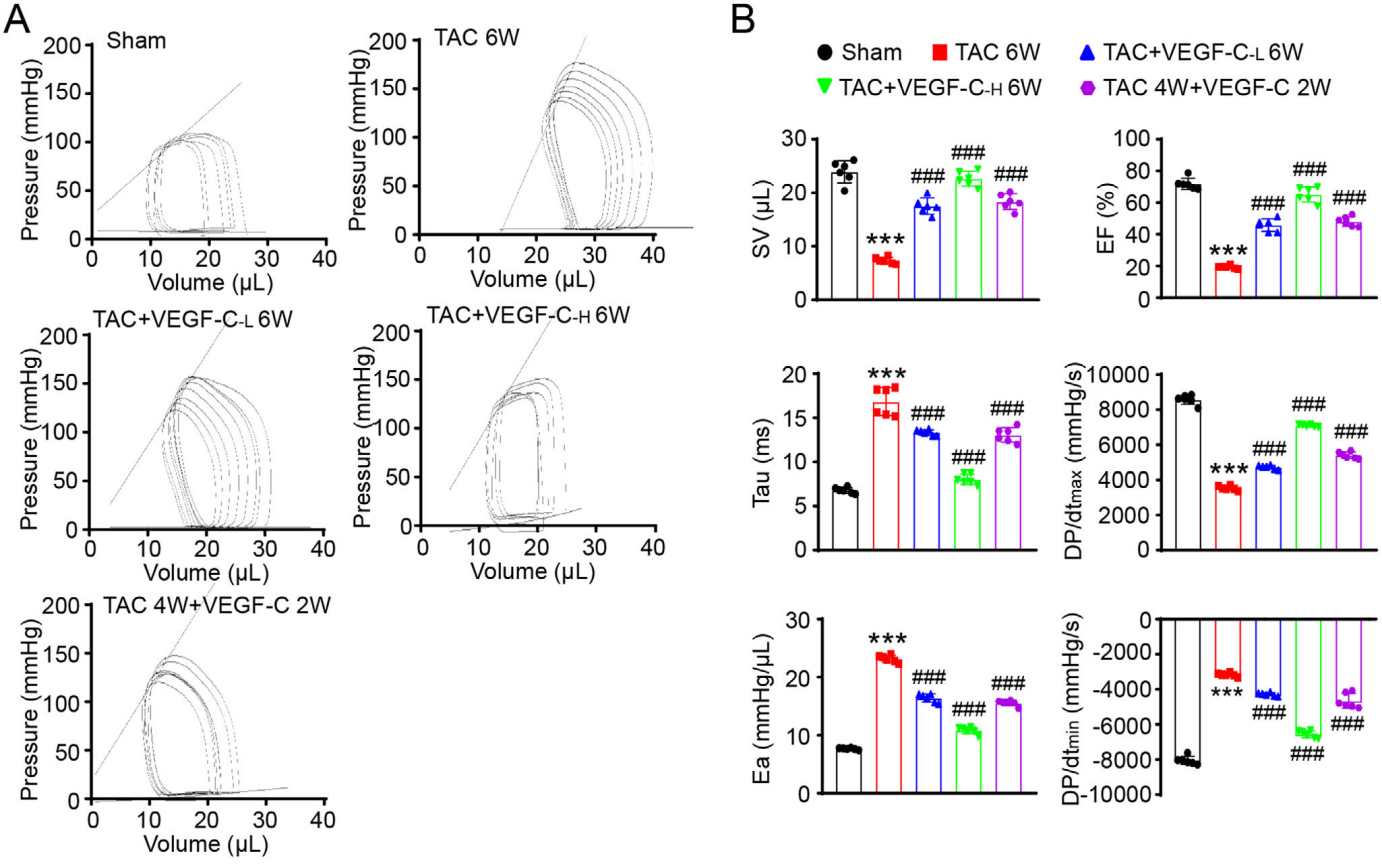
VEGF‐C_156S_ improves intact heart function after TAC. (A) In vivo heart function as shown by the LV pressure volume (PV) loops in control mice (sham), controls mice treated with 6 weeks of TAC with or without VEGF‐C_156S_ at doses of 33 and 100 ng/g (TAC+VEGF‐C_‐L_ 6W and TAC+VEGF‐C_‐H_ 6W), and mice with cardiac dysfunction induced by 4 weeks of TAC and then VEGF‐C_156S_ (100 ng/g) treatment for 2 additional weeks (TAC 4W + VEGF‐C_156S_ 2W). (B) Summary data on systolic function and diastolic function. The data are presented as the mean ± SD, and n represents the number of animals. Statistical analysis was performed with one‐way ANOVA; ***P < 0.001 versus sham; ^#^ *P *< 0.05 and ^###^ *P *< 0.001 versus TAC + saline

Finally, to confirm the cardioprotective effect of VEGF‐C_156S_ on heart function in vivo, we performed an invasive LV PV analysis with a conductance catheter. TAC for 6 weeks resulted in a marked rightward and upward shift of the PV loops and corresponding systolic and diastolic boundary relations. However, concurrent administration of VEGF‐C_156S_ dose‐dependently preserved cardiac volume and improved systolic function. VEGF‐C_156S_ treatment for the last 2 weeks, after hypertrophy was already established, also significantly improved cardiac dysfunction (Figure 9A). Consistently, TAC for 6 weeks greatly reduced cardiac performance and LV contractility, as indicated by a decrease in stroke volume, ejection fraction, and maximal rate of increase in LV pressure (dP/dt_max_). These effects were attenuated or eliminated by VEGF‐C_156S_ (Figure 9B, supplementary material online, Table S7). Similar results were also observed for diastolic dysfunction, as indicated by increased in the relaxation time constant (Tau) and arterial elastance (Ea; an index of total ventricular afterload) and a decreased in the maximal rate of decrease in LV pressure (dP/dt_min_) (Figure 9B). The results for additional functional variables are provided in the supplementary material online, Table S7.

## 4 DISCUSSION

This study demonstrates the importance of VEGF‐C‐VEGFR‐3 signaling in myocardial lymphangiogenesis, which is a novel regulatory pathway for the transition from pressure overload‐induced hypertrophy to HF. VEGFR‐3 knockdown aggravated pressure overload‐induced reductions in VEGF‐C‐VEGFR‐3 signaling and cardiac lymphangiogenesis, leading to cardiac edema, hypertrophic remodeling, and dysfunction, whereas administration of VEGF‐C_156S_ in mice had the opposite effect. Thus, our data support a causative role of the VEGF‐C‐VEGFR‐3 axis in the pathogenesis of cardiac remodeling and dysfunction after persistent pressure overload and highlight VEGF‐C_156S_ as a promising new candidate for the treatment of HF.

The VEGF family consists of at least five members, including VEGF‐A, VEGF‐B, VEGF‐C, VEGF‐D, and placental growth factor, which play crucial roles in the regulation of vascular growth and lymphangiogenesis in different tissues. VEGF‐C was the first identified VEGFR‐3 ligand and is required for the development of lymphatic vessels under both physiological and pathological conditions.^8^ Interestingly, the expression of VEGF‐C or VEGFR‐3 is markedly induced in different tissues and cell types in response to various stimuli. For example, VEGF‐C expression is not detectable in quiescent Pdpn‐expressing epicardial cells or CD68^+^ tissue‐resident macrophages in the adult heart but is induced in both cell types following MI.^18^ Moreover, the expression levels of VEGF‐C and VEGFR‐3 are markedly increased in the infarcted myocardium in animals,^17^, ^29^ and surviving CMs are the main sources of VEGF‐C and VEGFR‐3.^29^ However, the present results indicated that LECs are the main cell type expressing VEGF‐C and VEGFR‐3. Although our recent data demonstrate that angiotensin II markedly increases VEGFR‐3 expression and lymphangiogenesis in mouse LECs though the angiotensin type I receptor,^30^ the precise mechanisms by which pressure overload regulates VEGF‐C or VEGFR‐3 expression in LECs remain unclear. A previous study has shown that Tbx1, a member of the T‐box family of transcription factors, can upregulate VEGFR‐3 expression in endothelial cells by binding to an enhancer element within the Vegfr‐3 gene.^31^ This prompted us to examine Tbx1 expression in TAC‐operated hearts. We found that there were similar expression patterns between Tbx1 and VEGF‐C or VEGFR‐3 during the transition from adaptive cardiac hypertrophy to TAC‐induced HF (supplementary material online, Figure S4C), suggesting that Tbx1 may be involved in regulating VEGF‐C or VEGFR‐3 expression in the heart.

Chronic pressure overload elicits pathological hypertrophy associated with cardiac fibrosis and dysfunction.^1^, ^3^ Multiple molecular events and signaling pathways have been found to be involved in this process,^1^, ^3^ but the exact mechanisms need to be explored. Cardiac lymphatic dysfunction occurs in various cardiovascular diseases and influences myocardial fluid homeostasis and inflammation, which may induce vascular dysfunction, cardiac remodeling, and cardiac dysfunction after ischemic stress.^16^, ^18^, ^19^, ^20^, ^32^ In addition, VEGF‐C‐VEGFR‐3 signaling has been reported to be a key regulator of lymphatic vessels within different tissues. VEGF‐C and VEGF‐D are as potent stimulators of lymphangiogenesis that participate in cardiac tissue generation and tissue repair, and VEGF‐D can compensate for loss of VEGF‐C in some contexts.^33^ Conversely, inhibition of VEGF‐C/D signaling reduces infarct lymphangiogenesis and T‐cell infiltration but improves cardiac function post‐MI in animals.^34^ Furthermore, systemic blockade of VEGF‐C/D or VEGFR‐3 in db/db mice significantly attenuates obesity‐induced insulin resistance, adipose tissue M1 macrophage infiltration, and hepatic lipid accumulation.^35^ LYVE‐1 is highly expressed in lymphatic capillaries and is required for leukocyte clearance under inflammatory conditions. Deletion of Lyve1 in mice prevents docking and transmigration of leukocytes through the lymphatic endothelium, which exacerbates the chronic inflammatory response, leading to cardiac remodeling and dysfunction after MI.^18^ Furthermore, deficiency of LYVE‐1 in macrophages promotes arterial stiffening and collagen deposition,^36^ suggesting that LYVE‐1 is essential for active trafficking of leukocytes and optimal cardiovascular function. Similarly, our in vivo data indicated that LYVE‐1 dependent knockdown of VEGFR‐3 increased the overall macrophage load and promoted macrophage M1 polarization in TAC‐operated hearts, whereas the increased infiltration of macrophages was attenuated in the hearts of VEGF‐C_156S_‐treated mice, suggesting that impairment of lymphangiogenesis exacerbated cardiac remodeling partially by blocking macrophage clearance and polarization in VEGFR‐3 knockdown mice. In addition, VEGF‐C_156S_ has a non‐lymphatic effect on CMs, directly upregulates hypertrophic markers and reverses hypoxia‐induced CM atrophy.^37^ However, our in vitro data did not support a direct effect of VEGF‐C_156S_ on CM hypertrophy.

AKT/ERK1/2 and calcineurin/NFATC1/FOXC2 signaling are well‐characterized VEGFR‐2/3 downstream pathways that are crucial for regulation of lymphatic capillaries and the maturation of collecting lymphatic vessels.^8^, ^38^ Cx43 is a gap junction protein that is highly expressed in LECs and plays important roles in the regulation of cardiac lymphangiogenesis, edema, and contractile dysfunction after MI.^39^, ^40^ Interestingly, VEGFR‐3 knockdown significantly inhibited cardiac lymphangiogenesis and activation downstream mediators (AKT/ERK1/2, CaNA/NFATc1/FOX2C, and CX43) but did not affect cardiac function (FS%) in the sham‐operated groups, indicating that VEGFR‐3 knockdown is capable of maintaining cardiac water balance at baseline. However, upon TAC stress, VEGFR‐3 knockdown markedly inhibited VEGFR‐3‐mediated signals and cardiac lymphangiogenesis, leading to aggravation of cardiac edema, hypertrophy, and dysfunction, but these effects were markedly improved in VEGF‐C_156S_‐administered mice. This beneficial impact was similar to a protective effect against MI reported in a previous study.^16^ Taken together, these results indicate that VEGF‐C_156S_‐VEGFR‐3 axis‐dependent lymphangiogenesis is crucially involved in the transition from compensatory hypertrophy to HF and suggest that this pathway may be a promising target for the treatment of this disease.

Numerous growth factors, including angiopoietins, fibroblast growth factor‐2 (FGF2), hepatocyte growth factor, and VEGFs, have been identified to stimulate lymphangiogenesis.^41^, ^42^, ^43^, ^44^, ^45^ Native VEGF‐C can bind to VEGFR‐2 to increase angiogenesis and lymphatic vessel permeability, leading to lymphatic dysfunction.^44^, ^46^, ^47^ VEGF‐C_156S_ is a recombinant human VEGF‐C that selectively activates VEGFR‐3 in the lymphatic system.^12^, ^48^ Administration of VEGF‐C_156S_ can ameliorate lymphedema and acute lung allograft rejection and attenuate inflammatory bowel disease,^49^, ^50^ supporting its use as a potential therapy for different diseases. In recent years, the application of VEGF‐C_156S_ has expanded to the treatment of ischemic heart diseases such as MI and I/R injury in animals.^16^, ^17^, ^18^, ^19^ For example, VEGF‐C_156S_ treatment promotes cardiac lymphangiogenesis, which increases the clearance of inflammatory cells (myeloid cells, macrophages, and dendritic cells) and improves cardiac dysfunction in mice after MI in a LYVE‐1‐dependent manner.^17^, ^18^ Moreover, targeted intramyocardial delivery of VEGF‐C_152S_ (an analog of human VEGF‐C_156S_) using albumin‐alginate microparticles dose‐dependently enhances cardiac lymphangiogenesis and inhibits pre‐collecting lymphatic vessel remodeling, which improves myocardial edema, inflammation, fibrosis, and dysfunction in rats after MI.^16^ Similarly, stimulation of endogenous lymphangiogenesis with VEGF‐C_156S_ reduces myocardial inflammation and cardiac dysfunction after I/R injury,^19^ and administration of VEGF‐C via a local injection in Apo E^−/−^ mice can enhance lymphatic function and reduce the accumulation of cholesterol, which further suppresses atherosclerosis development.^51^ More recently, a study has demonstrated that VEGF‐C_156S_ treatment exerts multifaceted therapeutic effects to attenuate angiotensin II‐induced cardiac dysfunction by improving cardiac lymphatic function and reducing cardiac fibrosis, inflammation, and arterial hypertension.^52^ However, to the best of our knowledge, there have been no reports on the stimulation of cardiac lymphangiogenesis in a pressure overload setting. Thus, the previous findings led us to test whether therapeutic administration of VEGF‐C_156S_ protects against TAC‐induced HF in mice. Here, our novel evidence demonstrated that VEGF‐C_156S_ treatment selectively activated VEGFR‐3 and downstream signals in mouse LECs but not in other cell types. Moreover, systemic administration of VEGF‐C_156S_ effectively attenuated TAC stress‐induced cardiac edema and hypertrophy, thereby improving cardiac dysfunction as detected by echocardiography and invasive LV PV analysis. However, VEGF‐C_156S_ did not abolish the TAC‐induced cardiac hypertrophic response, indicating that VEGF‐C_156S_ markedly reduces cardiac edema and hypertrophy selectively through activation of VEGFR 3‐mediated lymphangiogenesis. Other mechanisms are also involved in this process. Together, these results strongly support the conclusion that VEGF‐C_156S_ is a promising new drug for the treatment of pressure overload‐induced cardiac dysfunction.

In conclusion, we have identified VEGF‐C‐VEGFR‐3 signaling as a critical regulator of pressure overload‐induced cardiac lymphangiogenesis, maladaptive hypertrophy, and HF. VEGF‐C_156S_ therapy is effective for improving cardiac edema, hypertrophy, and dysfunction. Thus, our findings are of clinical value given the high prevalence of hypertrophic heart disease, which plays an important role in volume or pressure overload‐induced HF. Further investigations are required to elucidate the molecular mechanism involved in the regulation of VEGF‐C and VEGFR‐3 expression in LECs and to test the effects of VEGF‐C‐VEGFR‐3 signaling in preventing cardiac hypertrophy and HF in other animal models.

## CONFLICT OF INTEREST

The authors have declared that there is no conflict of interest that could be perceived as prejudicing the impartiality of the research reported.

## AUTHOR CONTRIBUTIONS

Investigation: Qiu‐Yue Lin. Methodology: Qiu‐Yue Lin, Yun‐Long Zhang, and Jie Bai. Software: Qiu‐Yue Lin. Validation: Qiu‐Yue Lin. Analysis: Qiu‐Yue Lin. Data curation: Qiu‐Yue Lin, Yun‐Long Zhang, and Jie Bai. Supervision: Jin‐Qiu Liu and Hui‐Hua Li. Review and editing: Jin‐Qiu Liu and Hui‐Hua Li. Project administration and funding acquisition: Hui‐Hua Li.

## DATA AND MATERIALS AVAILABILITY

All data are included in the manuscript or in the supplementary material online.

## Supporting information

SUPPORTING INFORMATION

suppinfo1SUPPORTING INFORMATION

SUPPORTING INFORMATION

SUPPORTING INFORMATION

SUPPORTING INFORMATION

## Data Availability

The data for the current analysis are available upon reasonable request to the corresponding author.
