# Correction to VEGF‐C/VEGFR‐3 axis protects against pressure‐overload‐induced cardiac dysfunction through regulation of lymphangiogenesis

**DOI:** 10.1002/ctm2.1767

**Published:** 2024-07-12

The authors would like to change the image of LYVE‐1 staining in the sham group in previous Figure 1E as “RED square” in the below, which was published in Clinical and Translational Medicine. Lin QY, Zhang YL, Bai J, Liu JQ, Li HH. VEGF‐C/VEGFR‐3 axis protects against pressure‐overload‐induced cardiac dysfunction through regulation of lymphangiogenesis. Clin Transl Med. 2021;11(3):e374. https://doi.org/10.1002/ctm2.374).

**FIGURE 1:**
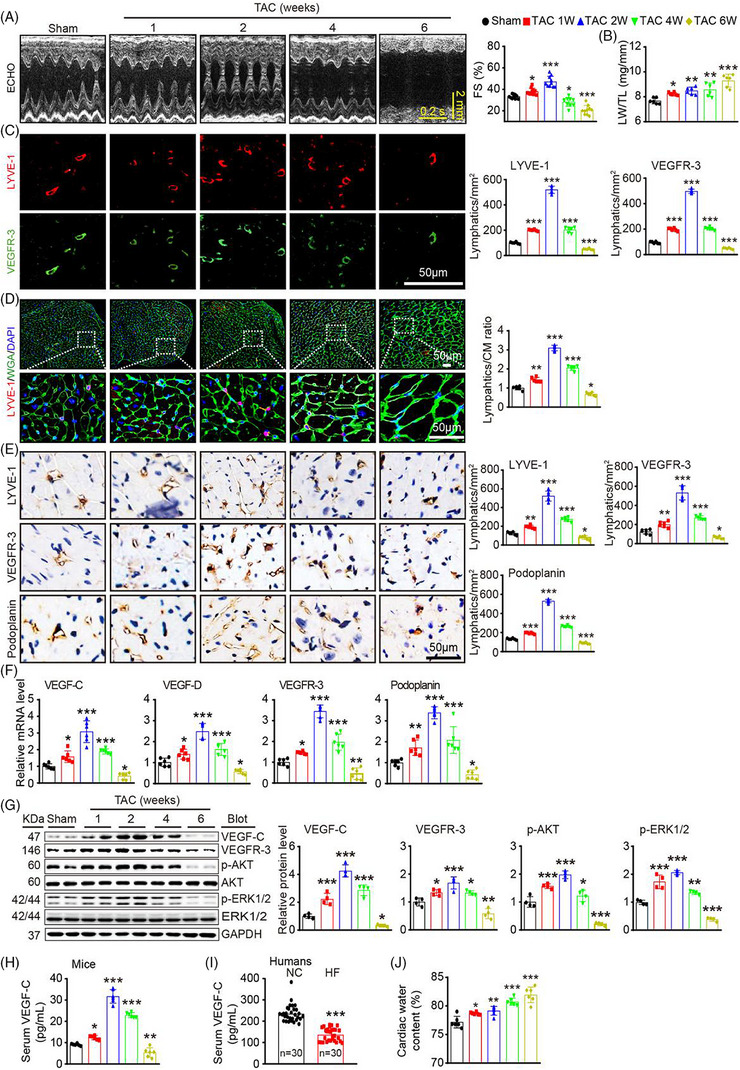
E is changed into the new image as “RED square” in the below.

