# Supplementary material for: VEGF‐C/VEGFR‐3 axis protects against pressure‐overload induced cardiac dysfunction through regulation of lymphangiogenesis
Source: Clin Transl Med. 2021 Mar 24;11(3):e374. doi: 10.1002/ctm2.374 (PMC7989711; doi:10.1002/ctm2.374)
Supplement: Supplementary file 1 — SUPPORTING INFORMATION [file CTM2-11-e374-s002.docx]

**Supplementary material online**

**Methods**

**Animals**

Wild-type (WT) C57BL/6 and Lyve-1^EGFP/cre^ mice ^1^ (hereafter referred to as Lyve-1^Cre^ mice) were purchased from The Jackson Laboratory (JAX strain number 012601). VEGFR-3-floxed (VEGFR-3^f/f^) mice ^2^ were purchased from The European Mouse Mutant Archive (EMMA) (ID: EM:09463). Allele PCR identification for Lyve-1^Cre^ and Vegfr-3^f/f^ mice was performed based on the genotyping protocols supplied by JAX and EMMA, respectively. To delete the VEGFR-3 gene in lymphatic endothelial cells, Lyve-1^Cre^ mice were mated with VEGFR-3^f/f^ mice to generate Lyve-1^cre^VEGFR-3^f/-^ (referred to as VEGFR-3^f/-^) mice because Lyve-1^cre^VEGFR-3^-/-^ was embryonic lethal.^3^ VEGFR-3^f/f^ mice were used as normal controls. Echocardiographic measurements showed that cardiac function (fractional shortening, FS%) was similar among the Lyve-1^cre^, VEGFR-3^f/f^ and VEGFR-3^f/-^ mice (n=10 per group, 8 weeks old) at baseline (Table S8), suggesting that the presence of Cre or flox has no effect on the development of cardiac function. Both VEGFR-3^f/f^ and VEGFR-3^f/-^ mice are viable and fertile in adulthood and were used in the current study on a C57BL/6J background.

**Echocardiography and pressure-volume (P-V) relationship assessment**

The mice were anesthetized with ketamine (0.2 g/kg) and xylazine (0.01 g/kg) by intraperitoneal injection and then subjected to transthoracic echocardiography at the indicated time points after transverse aortic constriction (TAC) surgery by using a 30 MHz probe (VisualSonics, Toronto, Ontario, Canada) as previously described.^4-6^ The indexes of ejection fraction (EF%), fractional shortening (FS%), left ventricular (LV) anterior wall thickness at diastole and systole (LVAW; d and LVAW; s, respectively), LV posterior wall thickness at diastole and systole (LVPW; d and LVPW; s, respectively), LV inner diameter at diastole and systole (LVID; d and LVID; s, respectively) were measured on the M-mode tracings and depended on at least three separate cardiac cycles. Blood pressure was measured in mice using a tail-cuff system (BP-2010A; Softron, Japan).

In vivo LV function was assessed by PV relationship measurement with a catheter (Millar system, ADINSTRUME NTS, Australia), as previously demonstrated.^7^ Briefly, mice were anesthetized with isoflurane (3%), and the right common carotid artery was isolated after making a cervical midline line skin incision. A 1.4 F PV catheter was gently cannulated through the right carotid artery until it entered the left ventricle for collection of pressure and volume signals. The parameters of the arterial elastance (Ea), the relaxation time constant (Tau), rate of pressure rise (+dP/dt) and rate of pressure decline (-dP/dt) were calculated with a PowerLab System (AD Instruments Pty Ltd., Mountain View, CA).

**Magnetic resonance imaging (MRI)**

MRI analyses of arterial spin labeling were performed in mice after anesthetization with ketamine (0.2 g/kg) and xylazine (0.01 g/kg) by intraperitoneal injection at 6 weeks post TAC, and the cardiac water content was evaluated by T2- mapping using a 4.7 T small animal magnet equipped with a horizontal bore scanner and an 86 mm internal diameter (Brucker), as previously described.^8^ Briefly, the anesthetized mice were placed in a warming holder. Two electrodes were placed subcutaneously on two sides of the chest to monitor the ECG signal, and MRI synchronization with cardiac rhythm was conducted with a small animal monitoring and gating system during only the systolic period. The T2 map images were generated and analyzed by ParaVision 5.1 software after exponential regression.

**Gravimetry**

Mice were anesthetized with ketamine (0.2 g/kg) and xylazine (0.01 g/kg) by intraperitoneal injection at 6 weeks post TAC. The mice were subjected to myocardial perfusion to remove the blood inside the heart with saline via a needle at a flow rate of 1 mL/min which had no effect on vascular permeability.^9^ Both the atria and great vessels were immediately removed to evaluate the wet weights of the left and right ventricles. The cardiac dry weight was obtained after desiccation of the ventricles for 5 days at 65 °C as previously described.^10^ The myocardial water content was calculated by the wet weight-dry weight method with the following equation: heart water content (%) = (wet heart weight - dry heart weight)/wet heart weight x 100 (%), similar to a method described previously.^11^

**Enzyme-linked immunosorbent assay (ELISA)**

ELISA was performed for quantitative measurement of VEGF-C in mouse serum. The concentrations were measured in a precoated 96-well plate with biotinylated detector antibodies specific for VEGF-C, IL-1β, IL-6 and TNF-α (Elabscience Biotechnology, Inc., Texas, USA) and MCP-1 (BOSTER) according to the manufacturer’s instructions. The relative OD450 for each test or standard well was calculated, and the VEGF-C concentration in each sample was interpolated via linear regression of the sample mean OD450 value relative to the standard curve.

**Plasma total** **cholesterol and triglyceride analysis**

Plasma samples from 8- to 10- week-old VEGFR-3^f/f^ and VEGFR-3^f/-^ male mice were collected and stored at low temperature. The CHOP-PAP method was used for quantitative measurement of the total cholesterol and triglyceride concentrations of each group with a cholesterol kit (BioSino) according to the manufacturer’s instructions. The concentrations in the samples were calculated against the standard curve at 505 nm.

**Mouse lymphatic endothelial cells (LECs) and culture**

Mouse LECs were purchased from Cellbio Company (CBR-131164). The LECs were cultured in basic DMEM medium supplemented with 10% FBS and 1% penicillin/streptomycin, as previously described.^12^ The LECs were pretreated with a selective VEGFR-3 inhibitor (20 nM, SAR131675, Selleck) or vehicle control and then stimulated with VEGF-C_156S_ (100 ng/mL) for 24 h after starvation.

**Rat cardiomyocyte (CM)** **isolation and culture**

Neonatal rat CMs (NRCMs) were isolated from Sprague-Dawley suckling rats within 24 h of birth as previously described.^13^ Briefly, the skin of neonatal rat was disinfected with 75% ethanol, and heart tissue was removed and cut into pieces. A mixture of trypsin and collagenase II was added to the heart pieces, which were then digested at 37 °C under shaking conditions. Signal cell suspensions were collected every 2 min, and digestion was stopped with DMEM/F-12 medium containing 2% FBS and 1% penicillin/streptomycin. Digestion was repeated serially until there was no visible mass of tissue. The signal cell suspensions were centrifuged after filtration. The cells were resuspended in DMEM/F-12 medium containing 15% FBS and then seeded in 100 mm plates. After 2 h of culture, the CMs were collected from the upper suspended medium, reseeded in a new plate and cultured for 24 h. The CMs were pretreated with a selective VEGFR-3 inhibitor (20 nM, SAR131675, Selleck) or vehicle control and then stimulated with VEGF-C_156S_ (100 ng/mL) for 24 h after starvation.

**Western blot analysis**

Total protein was extracted from fresh tissue or cultured cells by using Tissue Protein Extraction Reagent (Thermo Scientific, 78510) containing protease and phosphatase inhibitors according to the manufacturer’s instructions. Protein quantitation was performed by using a BCA Protein Assay Kit (Thermo Scientific, 23225). Briefly, 25-40 μg of total protein from each sample was separated via SDS-polyacrylamide gel electrophoresis and then transferred to 0.22 μm polyvinylidene fluoride (PVDF) membranes (Merck Millipore, ISEQ00010). The membranes were incubated with the indicated primary antibodies at 4 °C overnight after blocking with 5% defatted milk (BD Biosciences, 232100) in TBST buffer. The blots were detected with an automatic fluorescence chemiluminescence system (Fluor-Chem M, ProteinSimple, USA). The protein levels in the samples were normalized to those of GAPDH.

**Sample collection from patients with heart failure**

This study was performed in the First Affiliated Hospital of Dalian Medical University between January 2017 and May 2017. It included 30 patients with heart failure with EF% values <40% and 30 age- and sex-matched control subjects in a monocentric clinical cohort. Heart failure patients were diagnosed according to the 2016 ESC Guidelines.^14^ The baseline characteristics of the control subjects and patients are indicated in Table S1. Patients were not included if they had infectious disease, immunological disease, pulmonary disease, malignancies or any other infectious or immune diseases. The control subjects were defined as age- and sex-matched individuals with normal heart function and without any history of cardiac diseases or obvious abnormalities on physical examination (common checkup, clinical checkup, lab reports and B-ultrasonography reports) who did not meet the exclusion criteria. Written informed consent was obtained from each patient and control. The protocol conformed with the principles outlined in the Declaration of Helsinki and was approved by the Ethics Committee of the First Affiliated Hospital of Dalian Medical University. Blood samples were drawn into serum separator tubes (Becton Dickinson, UK), centrifuged and stored at -80 °C.

**References**

1. Pham THM, Baluk P, Xu Y, Grigorova I, Bankovich AJ, Pappu R, Coughlin SR, McDonald DM, Schwab SR, Cyster JG. Lymphatic endothelial cell sphingosine kinase activity is required for lymphocyte egress and lymphatic patterning. *J Exp Med* 2010;207:17-27.

2. Haiko P, Makinen T, Keskitalo S, Taipale J, Karkkainen MJ, Baldwin ME, Stacker SA, Achen MG, Alitalo K. Deletion of vascular endothelial growth factor C (VEGF-C) and VEGF-D is not equivalent to VEGF receptor 3 deletion in mouse embryos. *Mol Cell Biol* 2008;28:4843-4850.

3. Dumont DJ, Jussila L, Taipale J, Lymboussaki A, Mustonen T, Pajusola K, Breitman M, Alitalo K. Cardiovascular failure in mouse embryos deficient in VEGF receptor-3. *Science* 1998;282:946-949.

4. Wang X, Wang H-X, Li Y-L, Zhang C-C, Zhou C-Y, Wang L, Xia Y-L, Du J, Li H-H. MicroRNA Let-7i negatively regulates cardiac inflammation and fibrosis. *Hypertension* 2015;66:776-785.

5. Li N, Wang H-X, Han Q-Y, Li W-J, Zhang Y-L, Du J, Xia Y-L, Li H-H. Activation of the cardiac proteasome promotes angiotension II-induced hypertrophy by down-regulation of ATRAP. *J Mol Cell Cardiol* 2015;79:303-314.

6. Wang L, Li Y-L, Zhang C-C, Cui W, Wang X, Xia Y, Du J, Li H-H. Inhibition of Toll-like receptor 2 reduces cardiac fibrosis by attenuating macrophage-mediated inflammation. *Cardiovasc Res* 2014;101:383-392.

7. Chen C, Zou L-X, Lin Q-Y, Yan X, Bi H-L, Xie X, Wang S, Wang Q-S, Zhang Y-L, Li H-H. Resveratrol as a new inhibitor of immunoproteasome prevents PTEN degradation and attenuates cardiac hypertrophy after pressure overload. *Redox Biol* 2019;20:390-401.

8. Banquet S, Gomez E, Nicol L, Edwards-Lévy F, Henry J-P, Cao R, Schapman D, Dautreaux B, Lallemand F, Bauer F, Cao Y, Thuillez C, Mulder P, Richard V, Brakenhielm E. Arteriogenic therapy by intramyocardial sustained delivery of a novel growth factor combination prevents chronic heart failure. *Circulation* 2011;124:1059-1069.

9. Sanwal R, Khosraviani N, Advani SL, Advani A, Lee WL. The Endothelial Barrier Is not Rate-limiting to Insulin Action in the Myocardium of Male Mice. *Endocrinology* 2020;161.

10. Henri O, Pouehe C, Houssari M, Galas L, Nicol L, Edwards-Lévy F, Henry J-P, Dumesnil A, Boukhalfa I, Banquet S, Schapman D, Thuillez C, Richard V, Mulder P, Brakenhielm E. Selective Stimulation of Cardiac Lymphangiogenesis Reduces Myocardial Edema and Fibrosis Leading to Improved Cardiac Function Following Myocardial Infarction. *Circulation* 2016;133.

11. Luo Z, Diaco M, Murohara T, Ferrara N, Isner JM, Symes JF. Vascular endothelial growth factor attenuates myocardial ischemia-reperfusion injury. *Ann Thorac Surg* 1997;64:993-998.

12. Lin Q-Y, Bai J, Liu J-Q, Li H-H. Angiotensin II Stimulates the Proliferation and Migration of Lymphatic Endothelial Cells Through Angiotensin Type 1 Receptors. *Frontiers in Physiology* 2020;11:1122.

13. Wang L, Zhang Y-L, Lin Q-Y, Liu Y, Guan X-M, Ma X-L, Cao H-J, Liu Y, Bai J, Xia Y-L, Du J, Li H-H. CXCL1-CXCR2 axis mediates angiotensin II-induced cardiac hypertrophy and remodelling through regulation of monocyte infiltration. *Eur Heart J* 2018;39:1818-1831.

14. van der Meer P, Gaggin HK, Dec GW. ACC/AHA Versus ESC Guidelines on Heart Failure: JACC Guideline Comparison. *Journal of the American College of Cardiology* 2019;73:2756-2768.

**Table S1.** Baseline characteristics of normal control subjects and heart failure patients

| Parameters | Normal control  (n = 30) | Heart failure  (n = 30) | P-value |
| --- | --- | --- | --- |
| Age (years) | 52 ± 6.69 | 67 ± 13.96 | 0.000 |
| Male, n (%) | 30 | 80 | 0.000 |
| SBP (mmHg) | 112 ± 8.11 | 129 ± 21.59 | 0.000 |
| DBP (mmHg) | 70 ± 6.75 | 75 ± 13.86 | 0.107 |
| [Glu](javascript:;) (mmol/L) | 4.98 ± 0.38 | 5.84 ± 1.65 | 0.018 |
| Cre (μmol/L) | 60 ± 10.38 | 113 ± 62.13 | 0.000 |
| UA (μmol/L) | 308 ± 62.67 | 484 ± 215.85 | 0.000 |
| TC (mmol/L) | 5.21 ± 0.93 | 3.88 ± 1.18 | 0.000 |
| TG (mmol/L) | 1.43 ± 1.22 | 1.02 ± 0.83 | 0.111 |
| HDL (mmol/L) | 1.60 ± 0.27 | 0.86 ± 0.44 | 0.000 |
| LDL (mmol/L) | 2.79 ± 0.71 | 2.31 ± 0.74 | 0.012 |

SBP, systolic blood pressure; DBP, diastolic blood pressure; Glu, glucose; Cre, creatinine; UA, uric acid; TC, total cholesterol; TG, triglycerides; HDL, high-density lipoprotein; LDL, low-density lipoprotein.

**Table S2.** Primers used for quantitative real-time PCR analysis

| Gene | Forward primer (5’-3’) | Reverse primer (5’-3’) |
| --- | --- | --- |
| ANF | CACAGATCTGATGGATTTCAAGA | CCTCATCTTCTACCGGCATC |
| BNP | GAAGGTGCTGTCCCAGATGA | CCAGCAGCTGCATCTTGAAT |
| Collagen I | GAGTACTGGATCGACCCTAACCA | GACGGCTGAGTAGGGAACACA |
| Collagen III | TCCCCTGGAATCTGTGAATC | TGAGTCGAATTGGGGAGAAT |
| α-SMA | TCCTGACGCTGAAGTATCCGATA | GGCCACACGAAGCTCGTTAT |
| CD31 | AGCCAACAGCCATTACGGTTA | AGCCTTCCGTTCTCTTGGTG |
| Podoplanin | ACCCCAATAGAGATGGCTTGC | GGGCGAGAACCTTCCAGAAA |
| VEGFR-3 | CCGCAAGTGCATTCACAGAG | TCGGACATAGTCGGGGTCTT |
| VEGF-C | TGTGCTTCTTGTCTCTGGCG | CCTTCAAAAGCCTTGACCTCG |
| VEGF-D | CCTGGGACAGAAGACCACTC | TGAGATCTCCCGGACATGGT |
| IL-1β | TGCCACCTTTTGACAGTGATG | TGATGTGCTGCTGCGAGATT |
| IL-6 | TGATGGATGCTACCAAACTGGA | TGTGACTCCAGCTTATCTCTTGG |
| TNF-α | CAGGCGGTGCCTATGTCTC | CGATCACCCCGAAGTTCAGTAG |
| MCP-1 | TAAAAACCTGGATCGGAACCAAA | GCATTAGCTTCAGATTTACGGGT |
| Arg1 | CTCCAAGCCAAAGTCCTTAGAG | GGAGCTGTCATTAGGGACATCA |
| Ym1 | CAGGTCTGGCAATTCTTCTGAA | GTCTTGCTCATGTGTGTAAGTGA |
| IL-10 | CTTACTGACTGGCATGAGGATCA | GCAGCTCTAGGAGCATGTGG |
| GAPDH | GGTTGTCTCCTGCGACTTCA | GGTGGTCCAGGGTTTCTTACTC |

ANF, atrial natriuretic factor; BNP, brain natriuretic factor; α-SMA, α-smooth muscle actin; CD31, platelet and endothelial cell adhesion molecule 1; VEGFR-3, vascular endothelial growth factor receptor 3; VEGF-C, vascular endothelial growth factor C; VEGF-D, vascular endothelial growth factor D; IL-1β, interleukin 1 beta; IL-6, interleukin 6; TNF-α, tumor necrosis factor alpha; MCP-1, monocyte chemotactic protein 1; Arg1, arginase 1; Ym1, chitinase-like protein 3; IL-10, interleukin 10; GAPDH, glyceraldehyde 3-phosphate dehydrogenase.

**Table S3.** Echocardiographic parameters of wild-type mice after TAC surgery for 1 to 6 weeks or sham controls

|  |  | **TAC (weeks)** | | | |
| --- | --- | --- | --- | --- | --- |
|  | **Sham** | **1** | **2** | **4** | **6** |
| HR (bpm) | 543±31.47 | 552±18.09 | 546±7.40 | 538±9.65 | 531±51.67 |
| EF (%) | 63.44±2.56 | 70.96±5.70^*^ | 80.81±4.34^***^ | 48.26±7.04^***^ | 30.78±4.71^***^ |
| FS (%) | 33.23±1.73 | 38.02±2.81^*^ | 47.12±4.57^***^ | 28.29±3.66^*^ | 20.60±3.89^***^ |
| LVAW; d(mm) | 0.86±0.10 | 1.13±0.10^***^ | 1.32±0.07^***^ | 1.00±0.14^*^ | 0.73±0.05^*^ |
| LVPW; d(mm) | 0.71±0.11 | 0.92±0.10^***^ | 1.11±0.10^***^ | 0.78±0.10 | 0.60±0.05^*^ |
| LVID; d(mm) | 3.50±0.18 | 3.18±0.26^*^ | 2.83±0.22^***^ | 3.82±0.14^*^ | 3.97±0.27^***^ |
| LVAW; s(mm) | 1.35±0.09 | 1.63±0.09^***^ | 1.84±0.13^***^ | 1.20±0.09^*^ | 1.01±0.12^***^ |
| LVPW; s(mm) | 1.02±0.11 | 1.33±0.08^***^ | 1.54±0.07^***^ | 0.95±0.14 | 0.73±0.05^***^ |
| LVID; s(mm) | 2.29±0.11 | 1.83±0.20^**^ | 1.51±0.22^***^ | 2.75±0.30^**^ | 3.01±0.34^***^ |

Values: means ± SD (n = 10); ^*^p<0.05, ^**^p<0.01, ^***^p<0.001 vs. Sham;

HR, heart rate; EF, ejection fraction; FS, fractional shortening; LVAW; d, left ventricular anterior wall at end-diastole; LVPW; d, left ventricular posterior wall at end-diastole; LVID; d, left ventricular internal dimension at end-diastole; LVAW; s, left ventricular anterior wall at end-systole; LVPW; s, left ventricular posterior wall at end-systole; LVID; s, left ventricular internal dimension at end-systole.

**Table S4.** Echocardiographic parameters of wild-type (VEGFR-3^f/f^) and VEGFR-3 knockout (VEGFR-3^f/-^) mice after TAC surgery for 6 weeks or sham controls

|  | **Sham** | | **TAC (6w)** | |
| --- | --- | --- | --- | --- |
|  | **VEGFR-3^f/f^** | **VEGFR-3^f/-^** | **VEGFR-3^f/f^** | **VEGFR-3^f/-^** |
| HR (bpm) | 535±12.96 | 528±11.32 | 536±18.83 | 524±7.45 |
| EF (%) | 66.99±2.99 | 66.77±1.92 | 31.42±6.15^***^ | 24.44±3.64^###^ |
| FS (%) | 37.38±1.67 | 36.29±1.42 | 20.72±2.61^***^ | 13.18±2.05^###^ |
| LVAW; d(mm) | 0.87±0.04 | 0.87±0.03 | 0.75±0.03^***^ | 0.66±0.02^###^ |
| LVPW; d(mm) | 0.71±0.03 | 0.71±0.02 | 0.64±0.03^***^ | 0.58±0.02^###^ |
| LVID; d(mm) | 3.58±0.03 | 3.54±0.18 | 4.08±0.14^***^ | 4.71±0.15^###^ |
| LVAW; s(mm) | 1.36±0.02 | 1.38±0.03 | 1.05±0.03^***^ | 0.90±0.05^###^ |
| LVPW; s(mm) | 1.04±0.02 | 1.06±0.03 | 0.74±0.04^***^ | 0.63±0.03^###^ |
| LVID; s(mm) | 2.23±0.10 | 2.22±0.19 | 3.08±0.06^***^ | 3.93±0.14^###^ |

Values: means ± SD (n = 10); ^***^p<0.001 vs. VEGFR-3^f/f^ Sham; ^###^p<0.001 vs. VEGFR-3^f/f^ TAC;

HR, heart rate; EF, ejection fraction; FS, fractional shortening; LVAW; d, left ventricular anterior wall at end-diastole; LVPW; d, left ventricular posterior wall at end-diastole; LVID; d, left ventricular internal dimension at end-diastole; LVAW; s, left ventricular anterior wall at end-systole; LVPW; s, left ventricular posterior wall at end-systole; LVID; s, left ventricular internal dimension at end-systole.

**Table S5.** Echocardiographic parameters of wild-type mice treated with saline or VEGF-C_156S_ at doses of 33 (VEGF-C_-L_) and 100 (VEGF-C_-H_) ng/g daily after TAC surgery for 6 weeks or sham controls

|  |  | **TAC (6w)** | | |
| --- | --- | --- | --- | --- |
|  | **Sham** | **Saline** | **VEGF-C_-L_** | **VEGF-C_-H_** |
| HR (bpm) | 523±19.50 | 517±13.44 | 522±17.04 | 520±8.76 |
| EF (%) | 67.14±1.12 | 30.15±2.60^***^ | 42.10±1.59^###^ | 60.54±5.80^###^ |
| FS (%) | 36.28±1.70 | 19.28±3.43^***^ | 24.18±3.24^#^ | 31.17±3.62^###^ |
| LVAW; d(mm) | 0.89±0.05 | 0.72±0.02^***^ | 0.78±0.03^#^ | 0.86±0.04^###^ |
| LVPW; d(mm) | 0.76±0.03 | 0.61±0.02^***^ | 0.67±0.03^###^ | 0.74±0.02^###^ |
| LVID; d(mm) | 3.44±0.05 | 3.97±0.14^***^ | 3.81±0.09^##^ | 3.49±0.08^###^ |
| LVAW; s(mm) | 1.39±0.02 | 1.03±0.04^***^ | 1.20±0.03^###^ | 1.34±0.05^###^ |
| LVPW; s(mm) | 1.04±0.02 | 0.75±0.02^***^ | 0.83±0.02^###^ | 0.93±0.02^###^ |
| LVID; s(mm) | 2.20±0.10 | 3.25±0.16^***^ | 2.97±0.07^##^ | 2.55±0.20^###^ |

Values: means ± SD (n = 10); ^***^p<0.001 vs. Sham; ^#^p<0.05, ^##^p<0.01, ^###^p<0.001 vs. TAC + Saline;

HR, heart rate; EF, ejection fraction; FS, fractional shortening; LVAW; d, left ventricular anterior wall at end-diastole; LVPW; d, left ventricular posterior wall at end-diastole; LVID; d, left ventricular internal dimension at end-diastole; LVAW; s, left ventricular anterior wall at end-systole; LVPW; s, left ventricular posterior wall at end-systole; LVID; s, left ventricular internal dimension at end-systole.

**Table S6.** Echocardiographic parameters of wild-type mice after TAC for 4 weeks and then received saline or VEGF-C_156S_ (100 ng/g daily) for an additional 2 weeks

|  |  |  | **TAC** |  |
| --- | --- | --- | --- | --- |
|  | **Sham** | **4 W** | **6 W** | **6W+2 W VEGF-C** |
| HR (bpm) | 512±9.02 | 521±16.43 | 517±13.79 | 512±7.87 |
| EF (%) | 65.01±2.65 | 44.73±0.88^***^ | 30.69±4.84^###^ | 58.75±4.73^###$$$^ |
| FS (%) | 37.75±1.62 | 28.84±1.41^***^ | 19.55±1.11^###^ | 32.40±1.99^#$$$^ |
| LVAW; d(mm) | 0.86±0.03 | 1.00±0.04^***^ | 0.73±0.02^#^ | 0.83±0.02^###$$$^ |
| LVPW; d(mm) | 0.72±0.02 | 0.78±0.02^*^ | 0.59±0.04^###^ | 0.69±0.01^##$$$^ |
| LVID; d(mm) | 3.50±0.17 | 3.82±0.06^***^ | 4.01±0.05^#^ | 3.56±0.07^##$$$^ |
| LVAW; s(mm) | 1.36±0.03 | 1.21±0.02^***^ | 1.04±0.06^###^ | 1.30±0.03^##$$$^ |
| LVPW; s(mm) | 1.02±0.05 | 0.86±0.04^**^ | 0.72±0.03^##^ | 0.97±0.08^#$$$^ |
| LVID; s(mm) | 2.28±0.04 | 2.74±0.05^***^ | 3.03±0.03^###^ | 2.39±0.03^##$$$^ |

Values: means ± SD (n = 6 - 12); ^*^p<0.05, ^**^p<0.01, ^***^p<0.001 vs. Sham; ^#^p<0.05, ^##^p<0.01, ^###^p<0.001 vs. TAC 4 week; ^$$$^p<0.001 vs. TAC 6 week;

HR, heart rate; EF, ejection fraction; FS, fractional shortening; LVAW; d, left ventricular anterior wall at end-diastole; LVPW; d, left ventricular posterior wall at end-diastole; LVID; d, left ventricular internal dimension at end-diastole; LVAW; s, left ventricular anterior wall at end-systole; LVPW; s, left ventricular posterior wall at end-systole; LVID; s, left ventricular internal dimension at end-systole.

**Table S7.** Invasive left-ventricular pressure-volume parameters of wild-type mice in sham controls, controls treated with 6 weeks of TAC with or without VEGF-C_156S_, and mice with 4 weeks of TAC and then VEGF-C_156S_ added for additional 2 weeks

|  | |  |  | TAC | | | |
| --- | --- | --- | --- | --- | --- | --- | --- |
| Parameters |  | | **Sham** | **6W saline** | **6W VEGF-C_-L_** | **6W VEGF-C_-H_** | **4W+2W VEGF-C_-H_** |
| HR (bpm) | |  | 498±0.3 | 437±92 | 459±103 | 456±106 | 457±27 |
| SV (μL) | |  | 23.92±2.0 | 7.35±8.2^***^ | 17.51±6.7^###^ | 22.64±7.1^###^ | 18.34±1.3^###^ |
| CO (μL/min) | |  | 11919±905 | 3214±4244^*^ | 8029±3392^#^ | 10330±3427^#^ | 9761±462^#^ |
| EF (%) | |  | 72±3 | 19±26^***^ | 46±20^###^ | 65±21^###^ | 48±3^###^ |
| dP/dt max (mmHg/s) | |  | 8579±230 | 3545±2586^***^ | 4725±2150^###^ | 7106±2176^###^ | 5402±165^###^ |
| dP/dt min (mmHg/s) | |  | -8051±200 | -3161±2523^***^ | -4285±2444^###^ | -6550±2667^###^ | -4652±372^###^ |
| Ea (mmHg/μL) | |  | 7.7±0.1 | 23.2±8.1^***^ | 16.4±6.3^###^ | 10.9±5.9^###^ | 15.6±0.4^###^ |
| Tau (ms) | |  | 6.8±0.3 | 16.8±5.6^***^ | 13.3±4.4^###^ | 8.1±4.3^###^ | 13.1±0.7^###^ |
| Pmax (mmHg) | |  | 129±4 | 202±50^*^ | 167±44^#^ | 138±43^#^ | 148±3^#^ |
| SW (mmHg*μL) | |  | 2204±256 | 430±856^*^ | 914±681^#^ | 2557±918^#^ | 2797±208^#^ |

Values: means ± SD (n = 6); ^*^p<0.05 ^***^p<0.001 vs. Sham; ^#^p<0.05, ^###^p<0.001 vs. TAC 6W + saline;

HR, heart rate; SV, stroke volume; CD, cardiac output; EF, ejection fraction; dP/dt, peak rate of pressure decline; Ea, arterial elastance; Tau, relaxation time constant; Pmax, maximal power index; SW, slope of stroke work– end-diastolic volume relationship;

**Table S8.** Echocardiographic parameters of FS% for each individual mouse in LYVE-1^cre^, VEGFR-3^f/f^ and VEGFR-3^f/-^ mice in steady state conditions (n=10)

| LYVE-1^cre^ | VEGFR-3^f/f^ | VEGFR-3^f/-^ |
| --- | --- | --- |
| 36.40 | 37.72 | 37.82 |
| 38.79 | 38.36 | 36.46 |
| 37.72 | 38.39 | 34.04 |
| 30.56 | 36.85 | 35.99 |
| 38.39 | 36.61 | 36.46 |
| 39.85 | 34.19 | 37.04 |
| 38.33 | 35.56 | 36.28 |
| 34.19 | 38.55 | 36.91 |
| 36.72 | 36.37 | 37.62 |
| 37.04 | 37.19 | 37.33 |
| 36.80±2.43 | 36.98±1.25 | 36.59±0.97 |

**Figure legends**

**Figure S1.** **Cardiac hypertrophy, fibrosis, apoptosis, angiogenesis and macrophage infiltration during TAC.** WT mice were subjected to sham or TAC surgery and remained under sham or TAC conditions for 1 to 6 weeks. (A) Hematoxylin and eosin (H&E) staining of heart sections (left, scale bar: 0.50 mm) and the heart weight/tibial length (HW/TL) and heart weight/body weight (HW/BW) ratios (right, n = 6). (B) Tetramethylrhodamine (TRITC)-labeled wheat germ agglutinin (WGA) staining of heart sections (left, scale bar: 50 μm) and quantification of the myocyte cross-sectional area (right, 200 cells counted per heart, n = 6). (C) Masson's trichrome staining of heart sections (left) and quantification of the fibrotic area (right, n = 6). Scale bar: 50 μm. (D) qPCR analyses of ANF, BNP, α-SMA, collagen I and collagen III mRNA levels (n = 6). (E) Heart sections stained with TUNEL (red), α-actinin (green) and DAPI (blue) (left, scale bar: 50 μm) and quantification of TUNEL^+^ cardiomyocytes (CMs) (right, n = 6). (F) quantification of the Bax to Bcl-2 ratio (n = 4). (G) Heart sections immunostained with an anti-CD31 antibody (red) and DAPI (blue) (left, scale bar: 50 μm) and quantification of CD31^+^ vessels (right, n = 6). (H) qPCR analyses of CD31 mRNA levels (n = 6). (I) Heart sections stained with an anti-LYVE-1 antibody (red), an anti-CD68 antibody (green) and DAPI (blue) (left, scale bar: 50 μm) and quantification of the LYVE-1^+^, CD68^+^ and LYVE-1^+^CD68^+^ cells and the percentage of LYVE-1^+^CD68^+^ to LYVE-1^+^ cells (right, n = 6). The data are presented as the mean ± SD, and n represents the number of animals per group. Statistical analysis was performed with one-way ANOVA; **P*<0.05, ***P*<0.01 and *** *P*<0.001 vs. sham.

**Figure S2. Expression of VEGFR-3 and its effect on cardiac infiltration and polarization of macrophages, lipid levels, body weight and blood pressure in mice.** (A) Macrophages were derived from the bone marrow of VEGFR-3^f/f^ and VEGFR-3^f/-^ mice after 2 weeks of TAC. Immunoblot analysis of the VEGFR-3 protein in macrophages (left) and quantification of this protein (right, n = 4). GAPDH was used as an internal control. (B) Heart sections stained with an anti-CD68 antibody and DAPI (left) and quantification of CD68^+^ macrophages in the heart (right, n = 6). (C) Heart sections stained with an antibody against CD68 (red), CD86 or CD206 (green) and DAPI (blue) (left, scale bar: 50 μm) and quantification of CD68^+^CD86^+^ M1 macrophages or CD68^+^CD206^+^ M2 macrophages (right, n = 6). (D-E) qPCR analyses of IL-1β, IL-6, TNF-α, MCP-1, Arg1, Ym1 and IL-10 mRNA levels (n = 6). (F) ELISAs of the circulating cytokines IL-1β, IL-6, TNF-α and MCP-1 (n = 8). (G) Immunoblot analysis of the VEGFR-3 protein in the lungs, liver and intestines (upper) and quantification of this protein (lower, n = 4). GAPDH was used as an internal control. (H) Analysis of serum total cholesterol and triglyceride levels in VEGFR-3^f/f^ and VEGFR-3^f/-^ mice (n = 15-18). (I) Body weights (g) of VEGFR-3^f/f^ and VEGFR-3^f/-^ mice. (J) Systolic blood pressures (mmHg) of VEGFR-3^f/f^ and VEGFR-3^f/-^ mice. The data are presented as the mean ± SD, and n represents the number of animals per group. Statistical analysis was performed with Student’s t test; **P*<0.05 and ***P*<0.01 vs. VEGFR-3^f/f^ + TAC or Sham.

**Figure S3. Effect of VEGF-C_156S_ on cardiac lymphangiogenesis in mice after 6 weeks of TAC.** WT or VEGFR-3^f/-^ mice were injected with saline or VEGF-C_156S_ and subjected to TAC for 6 weeks. (A) Heart sections stained with an antibody against LYVE-1 (red) or VEGFR-3 (green) (left, scale bar: 50 μm) and quantification of LYVE-1^+^ or VEGFR-3^+^ lymphatic vessels (right, n = 6). (B) Heart sections stained with an anti-LYVE-1 antibody (red), TRITC-labeled WGA (green) and DAPI (blue) (left, scale bar: 50 μm) and the LYVE-1^+^ vessel to CM ratio (right, n = 6). (C) Gravimetric assessment of the cardiac water content (%) as determined by the cardiac dry weight to wet weight ratio (n = 6). The data are presented as the mean ± SD, and n represents the number of animals per group. Statistical analysis was performed with one-way ANOVA; ****P*<0.001 vs. TAC + saline; ^###^*P*<0.001 vs. TAC+VEGF-C-_H_.

**Figure S4. Effect of VEGF-C_156S_ on cardiomyocyte hypertrophy *in vitro* and the expression of** **Tbx1 during TAC.** (A) NRCMs were pretreated with the VEGFR-3 inhibitor SAR131675 or vehicle for 30 min and then stimulated with VEGF-C_156S_ or saline control for 24 h. Immunostaining of cells with an anti-α-actinin antibody (red) and DAPI (blue) (left, scale bar: 50 μm) and quantification of CM area (right, n = 6). (B) qPCR analyses of ANF and BNP mRNA levels (n = 6). (C) Immunoblot analyses of the Tbx1 proteins in the heart (left) and quantification of these proteins (right, n = 4). GAPDH was used as an internal control. The data are presented as the mean ± SD. Statistical analysis was performed with one-way ANOVA. **P*<0.05 and *** *P*<0.001 vs. sham.
