# Supplementary figures and images for: VEGF‐C/VEGFR‐3 axis protects against pressure‐overload induced cardiac dysfunction through regulation of lymphangiogenesis
Source: Clin Transl Med. 2021 Mar 24;11(3):e374. doi: 10.1002/ctm2.374 (PMC7989711; doi:10.1002/ctm2.374)

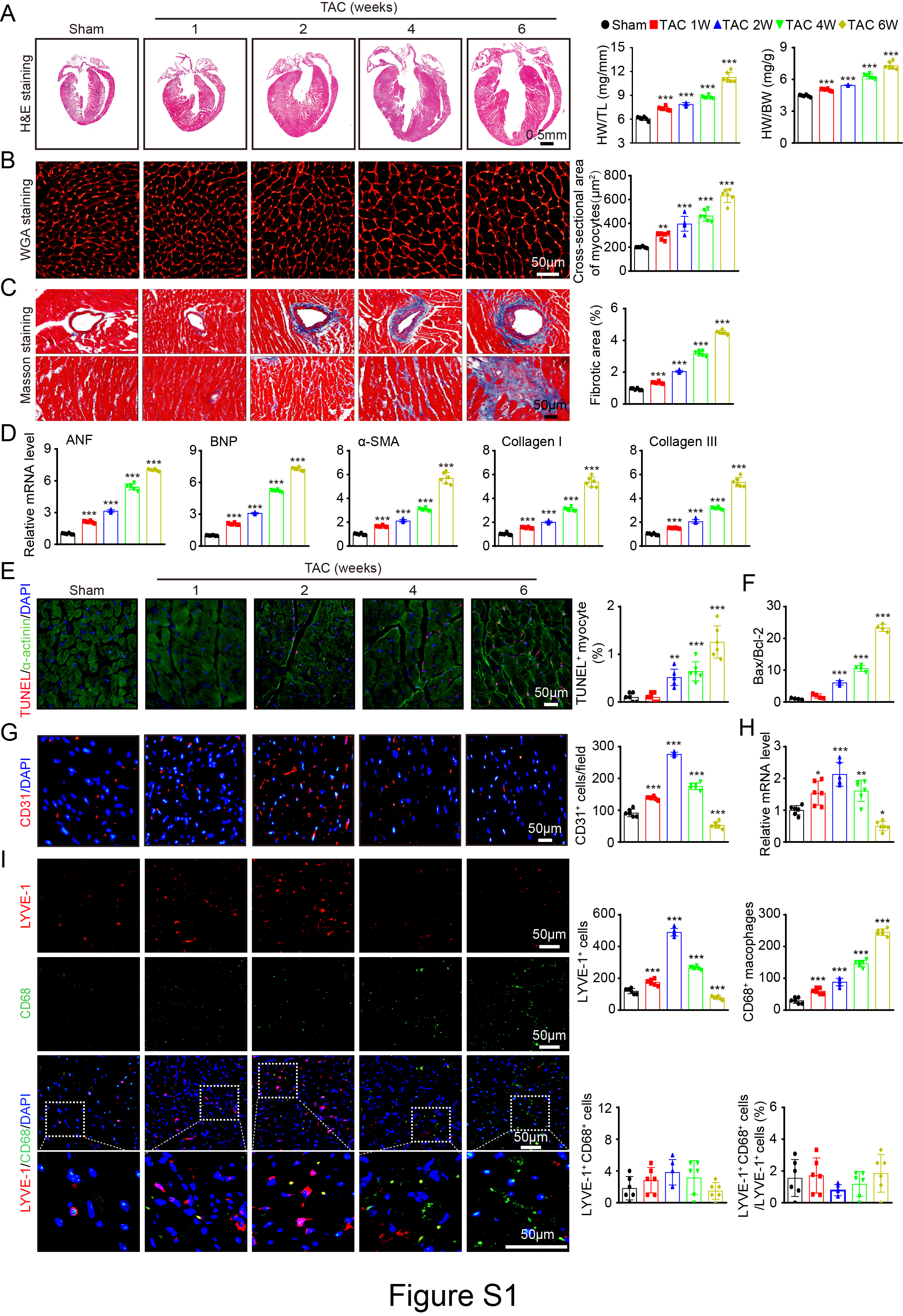

Supplement: Supplementary file 2 — suppinfo1SUPPORTING INFORMATION [file CTM2-11-e374-s004.jpg]

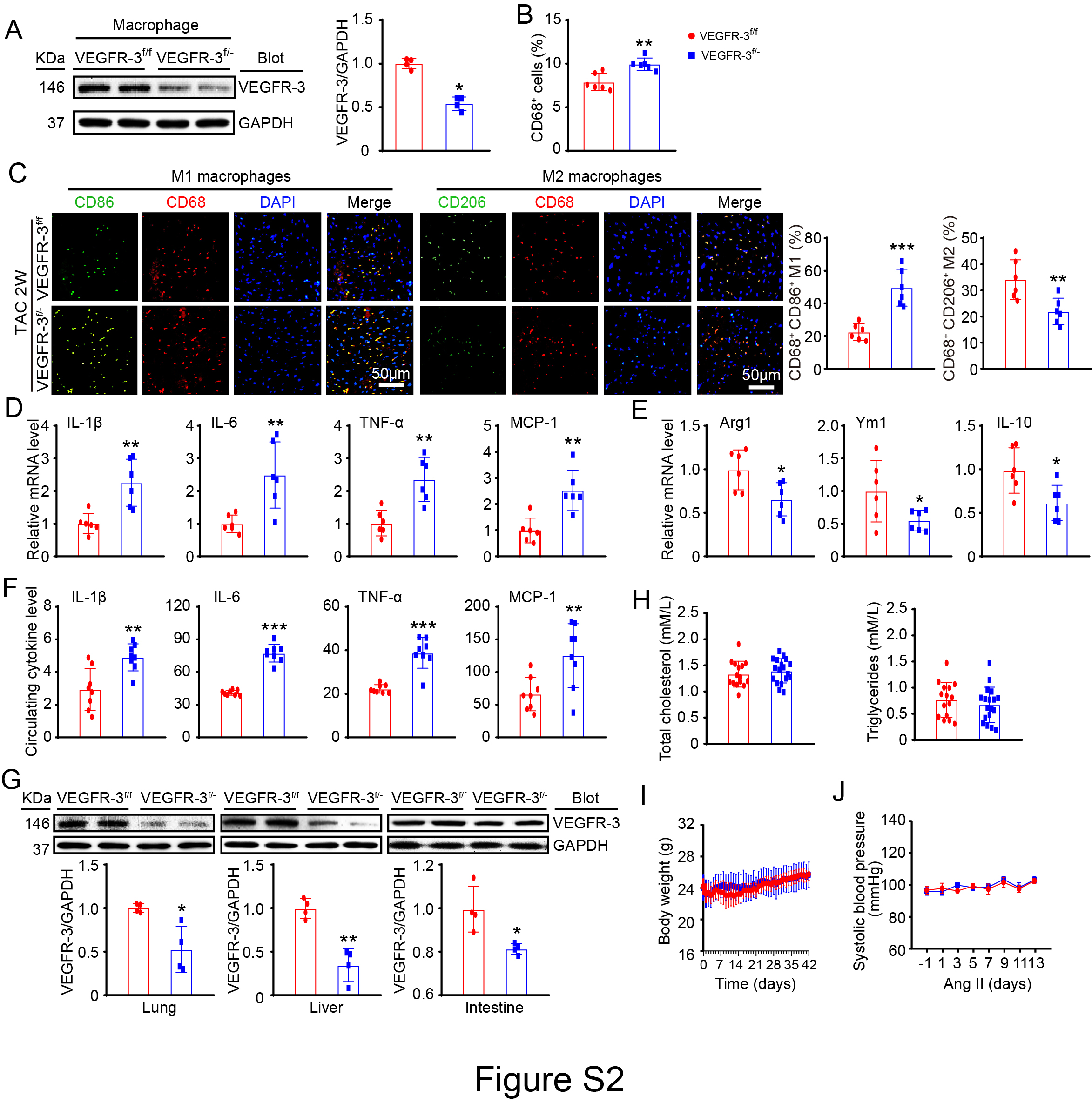

Supplement: Supplementary file 3 — SUPPORTING INFORMATION [file CTM2-11-e374-s001.jpg]

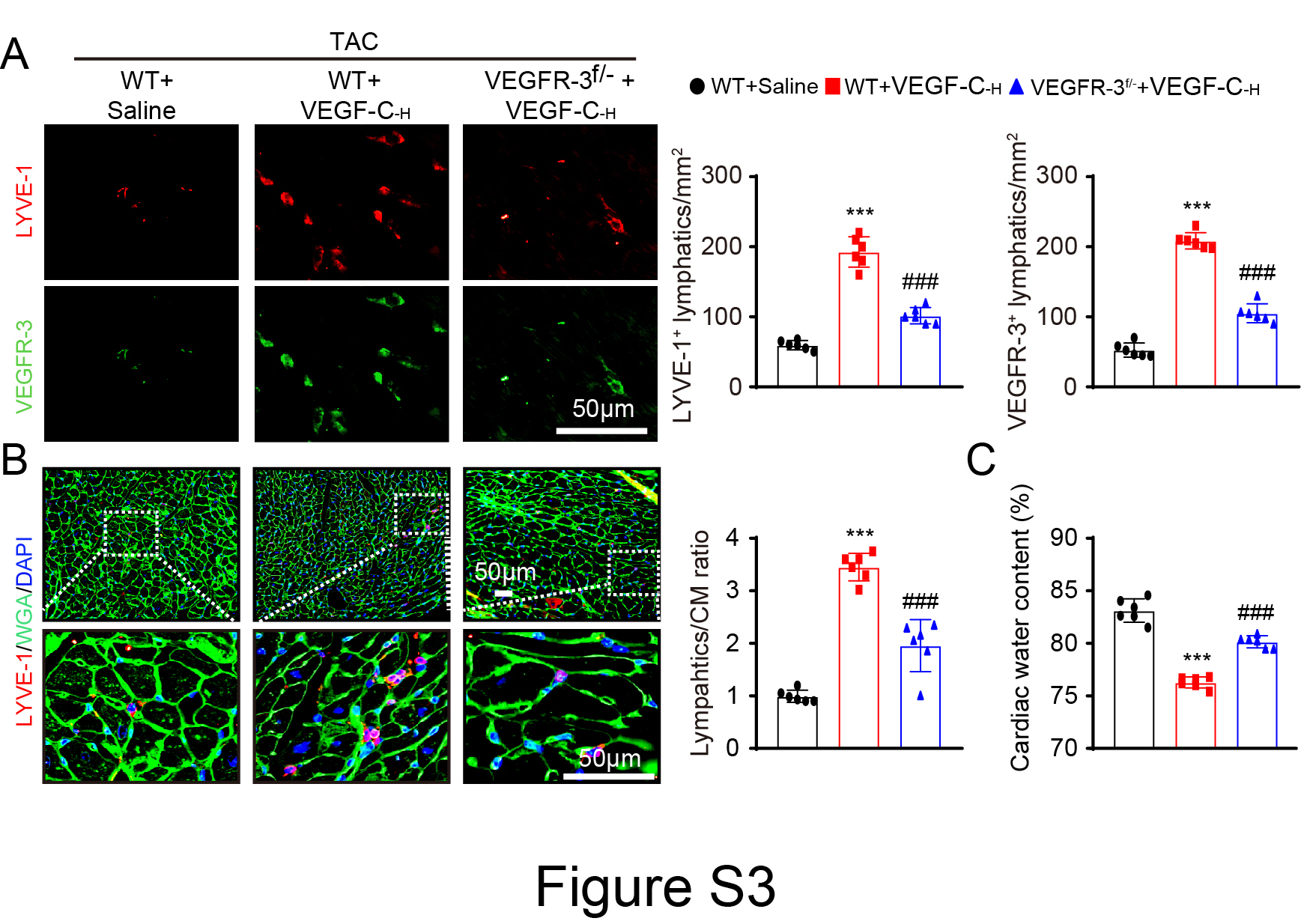

Supplement: Supplementary file 4 — SUPPORTING INFORMATION [file CTM2-11-e374-s005.jpg]

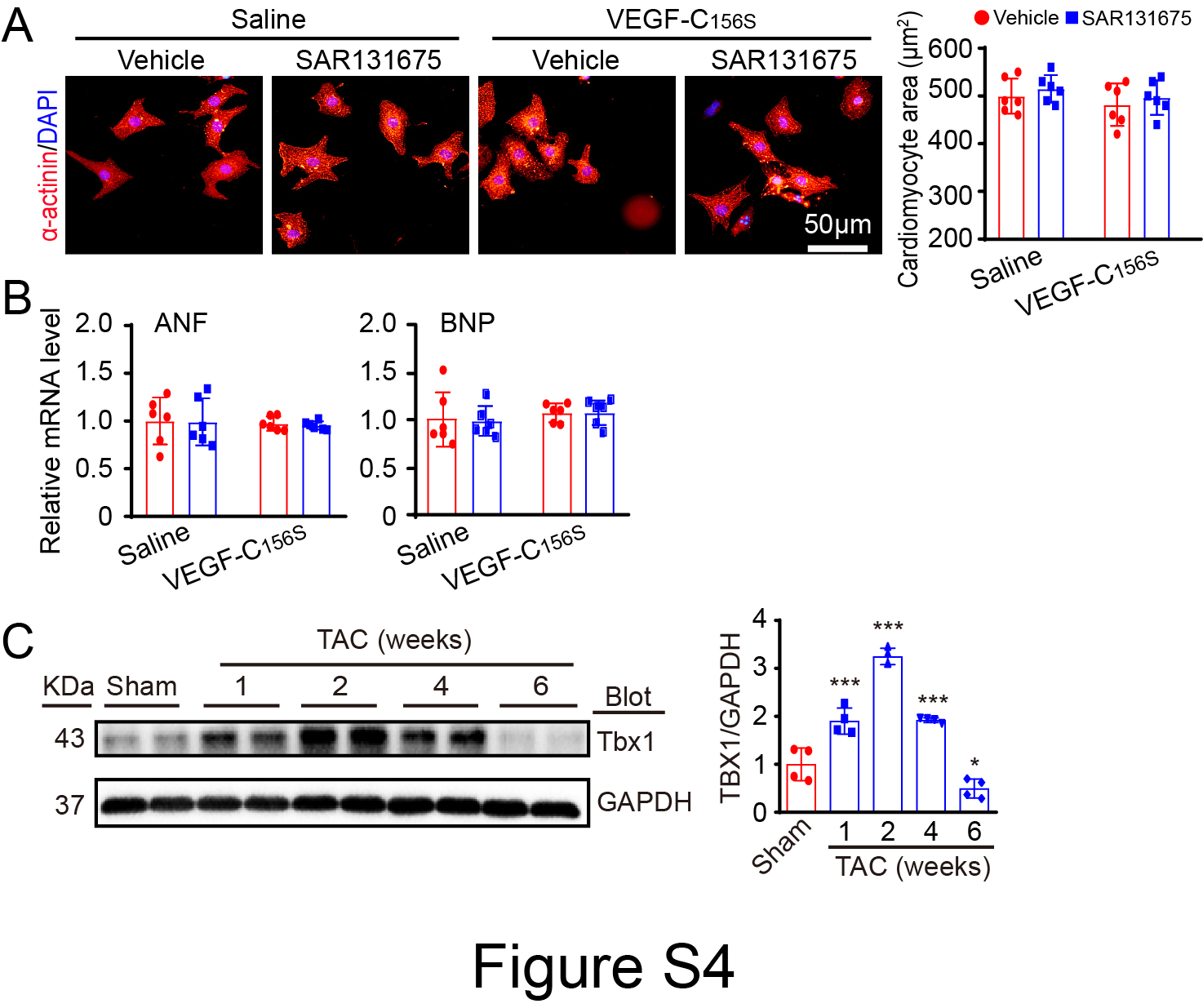

Supplement: Supplementary file 5 — SUPPORTING INFORMATION [file CTM2-11-e374-s003.jpg]
